# Harnessing nanozyme-immunomodulation for antiviral defense via Fe–nordihydroguaiaretic acid nano-networks

**DOI:** 10.1016/j.mtbio.2025.102567

**Published:** 2025-11-20

**Authors:** Hongping Wan, Zhengqun Huang, Mingrun Tang, Huirong Tan, Kai Deng, Yingnan Liu, Xinghong Zhao, Hongjun Chen

**Affiliations:** a Center for Infectious Diseases Control (CIDC), Sichuan Agricultural University, Chengdu, 611130, China; b State Key Laboratory of Veterinary Public Health and Safety, College of Veterinary Medicine, China Agricultural University, Beijing, 100193, China

**Keywords:** NA-Fe nano-networks, Lipoxidase-like activity, Antiviral activity, Innate immune responses, Comprehensive biosecurity

## Abstract

Enveloped viruses such as coronaviruses are highly transmissible, necessitating effective prevention and inactivation strategies. In this study, we engineered uniform, small-sized (∼57 nm) iron-phenolic nano-networks (NA-Fe) that exhibit high lipoxidase-like activity for the inactivation of enveloped virus, encompassing both DNA virus (Pseudorabies, PRV) and RNA virus (Transmissible Gastroenteritis, TGEV; and Porcine epidemic diarrhea virus, PEDV). Theoretical studies indicate that NA-Fe facilitates the adsorption of pentadiene moieties within the viral envelope through interactions between the *d*-orbital electrons of iron and the π-electrons of pentadiene. This interaction leads to activation of the C=C bonds, potentially resulting in disruption of the viral envelope. Besides its extracellular antiviral effects, NA-Fe also suppresses intracellular viral replication by stimulating antiviral innate immune responses, and upregulate the expression of interferon-stimulating genes (ISGs). *In vivo* studies demonstrate that NA-Fe exhibits favorable biosafety, and intranasal administration significantly reduces viral titers while providing effective antiviral protection in PRV-infected mice. Notably, surfaces coated with NA-Fe, such as fences and air purifiers in farm facilities, provide effective antiviral protection. Collectively, these findings demonstrate that NA-Fe exhibits potent broad-spectrum antiviral activity against both extracellular and intracellular viruses, highlighting its potential for application in comprehensive biosecurity and infectious disease control.

## Graphical abstract

**Figure:**
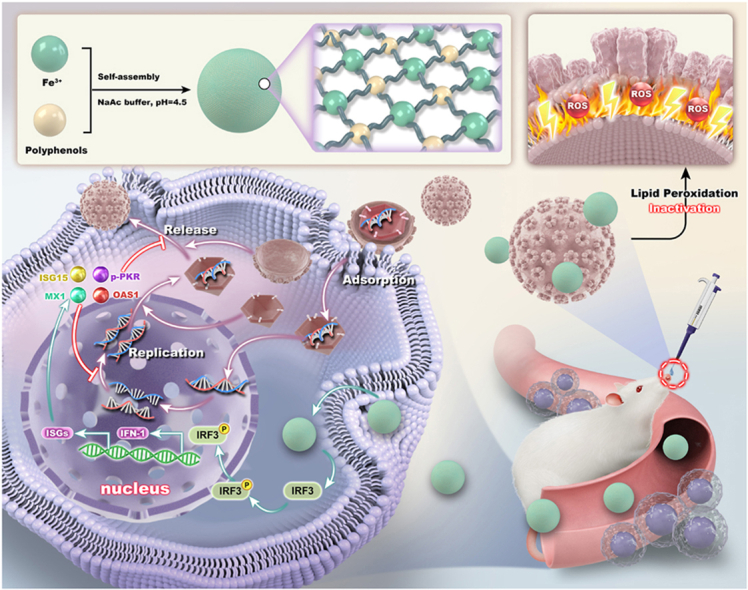

## 1 Introduction

Viral outbreaks pose a significant threat to public health. Due to their high mutation rates, viruses often evade vaccine-induced immunity, and the limited number of available antiviral drugs are typically virus-specific [1]. Consequently, there is an urgent need to develop broad-spectrum antiviral therapies capable of combating emerging viral threats. With the advancement of nanotechnology, various inorganic nanomaterials, such as titanium dioxide [2], gold [3], silver [4], and carbon quantum dots [5], have demonstrated promising antiviral potential. These nanoparticles primarily exert antiviral effects through two mechanisms: (1) direct disruption of the viral envelope [6,7], and (2) generation of reactive oxygen species (ROS) via metal ion release [8], which inactivates both viruses and host cells. However, most research has focused on extracellular virus inactivation, and their ability to combat a broad range of viruses remains limited. As a result, clinically applicable antiviral nanomaterials are still scarce.

Nanozymes, synthetic nanomaterials with enzyme-like catalytic activity, have emerged as promising candidates in antiviral research [[9], [10], [11]]. These materials typically exhibit one or more of peroxidase, catalase, or lipoxidase-like activity. Compared with natural enzymes, nanozymes have the advantages of high stability, long storage time and low cost, and have broad application prospects in biotherapy, biosensing, bioanalysis, antiviral treatment and environmental remediation [12]. Recent studies have shown that the antiviral activity of nanozymes is closely related to their lipoxidase-like activity. Qin et al. [13] demonstrated that traditional iron oxide (Fe_3_O_4_) nanozymes could inactivate enveloped influenza viruses via lipoxidase-like activity. Later on, they developed FeS nanozymes with enhanced lipoxidase-like activity [11], which exhibited antiviral efficacy against multiple influenza strains. Li et al. [9] further synthesized a diatomic iron nanozyme (Fe_2_DAC) with high lipoxidase-like activity, showing that it could inactivate various enveloped viruses by oxidizing their lipid membranes. These findings collectively underscore the potential of iron-based nanozymes in antiviral applications. Despite these advances, current nanozymes face several limitations, including large particle sizes (>100 nm), heterogeneous morphology, limited stability, unclear *in vivo* metabolic pathways, and insufficient biosafety evaluations. These limitations have hindered their further application in biomedical engineering. Most studies remain confined to *in vitro* virus inactivation. Although FeS nanozyme decoctions have demonstrated the ability to inhibit intracellular viral replication [11], the undefined composition of these formulations has hindered their translation for *in vivo* applications. Therefore, the development of nanozymes with enhanced lipoxidase-like activity, optimized physicochemical properties, and high biocompatibility holds substantial potential for broad-spectrum antiviral therapy. Natural polyphenolic compounds—such as [[14], [15], [16]], resveratrol [17], and nordihydroguaiaretic acid [18,19]—have been reported to inhibit viral replication *in vivo*. Additionally, numerous studies have shown that the introduction of polyphenols can enhance enzymatic activity of iron-based nanozymes [20,21]. However, the antiviral capability of these nanozymes remains largely undiscovered.

**Scheme 1:**
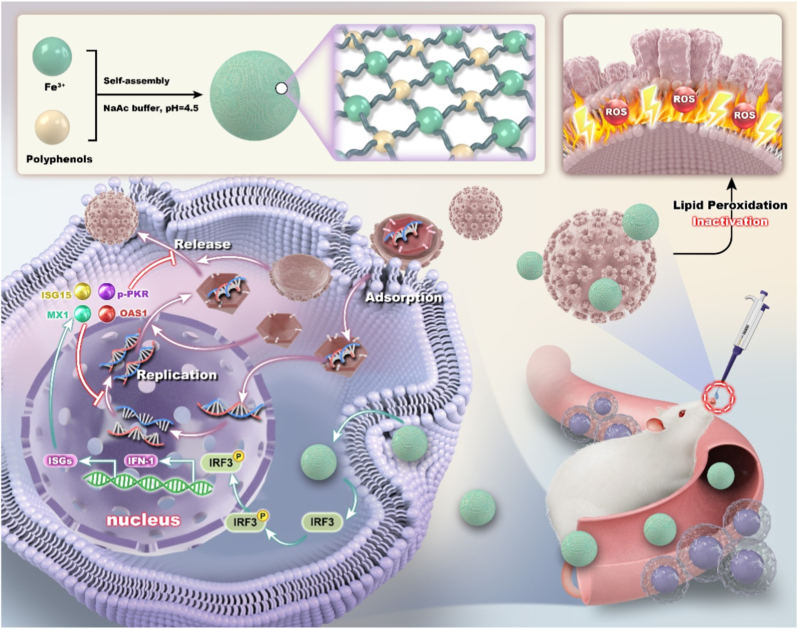
Schematic illustration of the antiviral activity and mechanism of NA-Fe. For extracellular viruses, NA-Fe exhibits high lipoxygenase-like activity, which disrupts the viral envelope, leading to virus inactivation. For intracellular viruses, NA-Fe stimulates the host's innate immune response, thereby suppressing viral replication and release.

From the above considerations, a library of Fe-phenolic nano-networks is designed and synthesized by a one-pot method. Among them, nordihydroguaiaretic acid–Fe (NA-Fe) nano-network with optimal enzymatic activity, good stability, uniform morphology, and small particle size (∼57 nm) was screened out. After elucidating the chemical structure of NA-Fe by using synchrotron radiation and high-resolution mass spectrometry. Its biosafety, broad-spectrum antiviral activity, and potential biomolecular mechanisms of antivirus both *in vitro* and *in vivo* were verified by using three different virus strains (PRV, TGEV, and PEDV), PK15 cells, and mice with or without infected by PRV (Scheme 1). Theoretical calculations by using density functional theory (DFT) and density of states (DOS) analysis further elucidate the physicochemical mechanism underlying the catalytic viral inactivation. Finally, as a proof of concept, NA-Fe was integrated into air purifier filters and farm facility fences to verify their antiviral activity *in situ*. Our findings indicate that NA-Fe exhibits potent broad-spectrum against both extracellular and intracellular viruses, supporting its potential for application in comprehensive biosafety and infectious disease control.

## 2 Results

### 2.1 Synthesis and characterizations of NA-Fe nano-networks

**Fig. 1:**
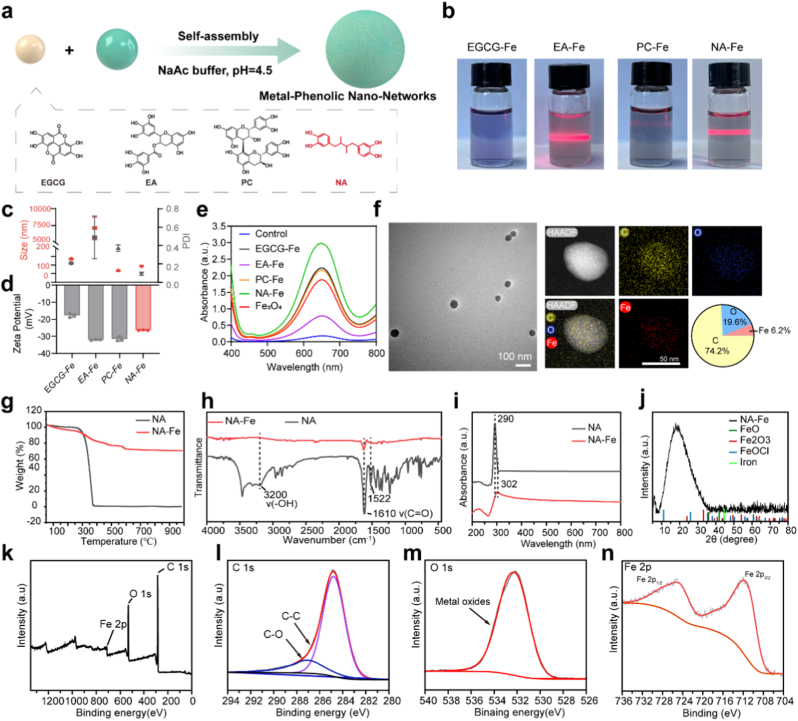
Synthesis and characterizations of NA-Fe. a) Schematic illustration of the preparation of metal-phenolic nano-networks. b) Representative pictures showing the Tyndall effect of metal-phenolic nano-networks (1 mg/mL). c) Diameter distribution of the various metal-phenolic nano-networks. d) Zeta potentials of various metal-phenolic nano-networks. e) UV–vis absorption of TMB after the treatments with different metal-phenolic nano-networks for 10 min in the presence of 1 mM H_2_O_2_. f) TEM image and TEM mapping of NA-Fe. g) TGA of NA and NA-Fe. h) FT-IR spectra of NA and NA-Fe. i) UV–vis absorption spectrum of NA and NA-Fe. j) XRD patterns of NA-Fe. k) XPS full-scan spectra of NA-Fe. l) C 1s, m) O 1s, n) Fe 2p XPS spectra of NA-Fe.

To prepare the phenolic-Fe nano-networks, various natural phenols were mixed with Fe^3+^ in an acidic solution (pH 4.5) at ambient temperature, in which phenols act as the reducing and chelating agent. The entire preparation process was facile and environmentally friendly. Representative natural phenols such as epigallocatechin gallate (EGCG), ellagic acid (EA), Proanthocyanidins (PC), and Nordihydroguaiaretic acid (NA) were used to study the structure-activity relationship of the formed nano-networks with the synthesis scheme shown in Fig. 1a. Among the formed nano-networks, EA-Fe and NA-Fe exhibited a distinct Tyndall effect (Fig. 1b) upon laser irradiation, implying the formation of fine nanoparticle [22,23]. In contrast, this phenomenon was not observed in EGCG-Fe and PC-Fe. Subsequent dynamic light scattering (DLS) indicated that the as-prepared phenolic/Fe nanonetworks varied greatly in hydrodynamic diameters (Fig. 1c and d). Still, they have negatively charged surfaces with zeta potential ranging from −17.7 mV to −32.1 mV. With all nano-networks in hand, we first evaluated their peroxidase-like activity by Fenton-like activity reaction with H_2_O_2_ and 3,3″,5,5″-tetra-methylbenzidine (TMB), whose blue-colored oxidation product can be assayed by measuring the absorbance at 652 nm [24]. The results in Fig. 1e and Fig. S1 showed that NA-Fe exhibits darkest blue coloration, indicating the highest peroxidase-like activity among all nano-networks. The catalytic kinetics of NA-Fe followed typical Michaelis-Menten kinetics. The Vmax values to H_2_O_2_ and TMB were 166.1 nM/s and 265.9 nM/s (Fig. S2, Tables S1-2), respectively, significantly exceeding those of Fe_3_O_4_, a representative iron-based nanozyme. Furthermore, no significant changes in particle size, zeta potential,and POD-like activity were observed in NA-Fe nano-networks over a 28-day observation period following exposure to PBS (Fig. S3), indicating that NA-Fe nano-networks possess excellent physicochemical stability. Previous studies have demonstrated that the morphology, size, and surface chemistry of nanoparticles play critical roles in determining their catalytic performance [25]. The NA-Fe nano-networks exhibit high nanozyme activity, which may be attributed to their small and uniform particle size, as well as their excellent structural stability. Considering the distinct Tyndall effect, high nanozyme activity, small hydrodynamic diameter, and excellent stability, NA-Fe nano-networks were selected in our subsequent studies. First, we characterized NA-Fe with transmission electron microscope (TEM) and dynamic light scattering (DLS). The results revealed that the NA-Fe nano-networks possess a spherical morphology with an average diameter of 57 nm and a polydispersity index (PDI) of 0.086 (Fig. 1c and f and Figure S4). Elemental mapping further confirmed that NA-Fe was composed of carbon (74.2 %), oxygen (19.6 %), and iron (6.2 %), which were homogeneously distributed over the entire nanoparticle. Subsequent, thermogravimetric analysis (TGA) recorded the less weight loss of NA-Fe compared to NA with increasing temperature (Fig. 1g), indicating coordination between Fe^3+^ and NA. Fourier transform infrared (FTIR) spectra of NA and NA-Fe were shown in Fig. 1h. The peaks at ≈3200 and 1610 cm^−1^ were assigned to the O–H and C=O stretching vibrations (Fig. 1h), respectively. The peak resulting from C=O exhibited a blue-shift after Fe doping in NA-Fe (1522 cm^−1^) could be caused by the Fe chelated with O [20]. Similarly, a weakened and red-shifted absorption peak around 302 nm was observed in NA-Fe nano-networks compared to NA by UV–vis (Fig. 1i), suggesting the phenolic groups chelated with Fe. The structure of NA-Fe nano-networks was also characterized by X-ray diffraction (XRD), in which a wide peak in the spectrum was observed, indicating a unique crystalline structure of NA-Fe nano-networks, which was different from other Fe-based materials (i.e., FeO, Fe_2_O_3_, Iron, and FeOCl) (Fig. 1j). Additionally, X-ray photoelectron spectroscopy (XPS) was further performed to explore the composition of NA-Fe nano-networks (Fig. 1k–n). A full-scan spectrum confirmed that NA-Fe nano-networks mainly consisted of elements of carbon, oxygen, and iron (Fig. 1k). From high resolution C 1s spectra, two peaks were observed at 284.8 and 287 eV (Fig. 1l), which can be ascribed to C-C (35.8 %), and C = O (8.41 %) species, respectively. The O 1s XPS spectrum showed an obvious metal oxides peak at 532.2 eV (Fig. 1m). The Fe 2p spectrum showed two peaks at 711.6 and 724.4 eV, ascribed to the Fe 2p3/2 and Fe 2p1/2 of Fe (II), confirmed the chelation of Fe chelated with phenolic groups in NA-Fe nano-networks (Fig. 1n).

**Fig. 2:**
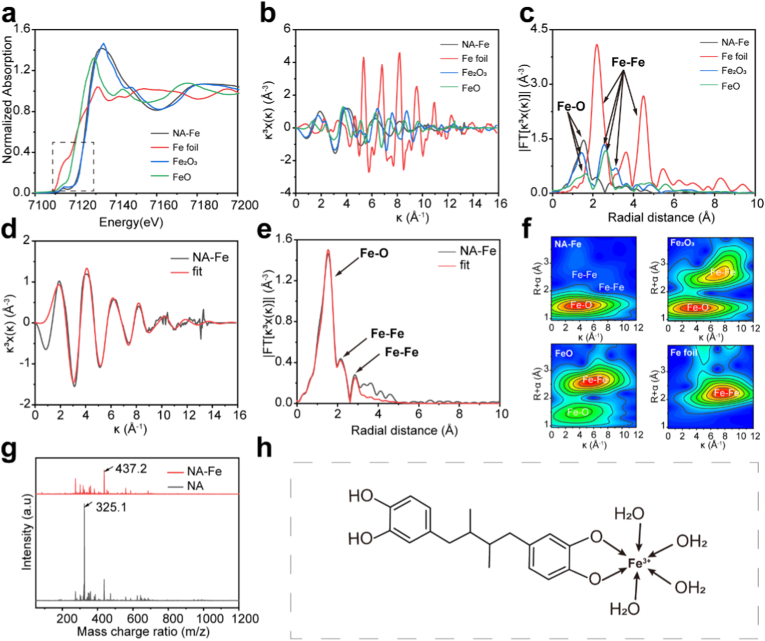
Determination of the coordination mode of Fe on NA. a) Fe k-edge XANES spectra of NA-Fe and reference samples. b) The magnitude of the Fourier transforms of the k3-weighted EXAFS and c) the corresponding EXAFS oscillation spectra of Fe-RA and reference samples at k space. d) EXAFS fitting result in R space. e) EXAFS fitting result at k space. f) Fe k-edge wavelet transform for k^3^-weighted EXAFS signals of NA-Fe and reference samples., g) HRMS for NA and NA-Fe. h) The configuration of NA-Fe with a coordination center of Fe^3+^.

To determine the chemical state and coordination environment of the iron sites in NA-Fe, synchrotron X-ray absorption spectroscopy (XAS) of Fe *k*-edge was performed on NA-Fe. From the Fe *k*-edge X-ray absorption near-edge structure spectra (XANES) in Fig. 2a, it can be seen that the absorption energy of Fe *k*-edge in Fe-RA is close to Fe_2_O_3_ (within the dashed box). This indicates that the valence state of iron in NA-Fe may mainly be +3. The extended X-ray absorption fine structure spectrum (EXAFS) shows that the oscillation peak in NA-Fe is ~ 1.5 Å (Fig. 2b and c), and the peak shape at this point is like Fe_2_O_3_, indicating an Fe-O coordination. The Fourier transform of the *k*^3^-weighted EXAFS for NA-Fe, along with references and the corresponding EXAFS fitting curves in k space (Fig. 2d and e), reveals Fe-O and Fe-Fe signals in NA-Fe.

Theoretically, Fe–Fe coordination is possible due to the strong reducing ability of phenolic groups [26,27]. However, EXAFS data fitting results for NA-Fe (Table S3) indicate a Fe–Fe coordination number of 1.8, substantially lower than that of iron (coordination number = 12), suggesting that the Fe–Fe content in NA-Fe is extremely low. In further wavelet transform of NA-Fe and references in *k* space (Fig. 2f) demonstrate the predominant coordination mode of Fe elements in NA-Fe is Fe-O coordination, rather than Fe-Fe coordination.

In addition to EXAFS results, the coordination configuration in NA-Fe is also calculated by using high resolution mass spectrum (HRMS). As shown in Fig. 2g, the *m*/*z* 325.1 peak corresponds to the molecular of NA-Fe. Combined with EXAFS data fitting results, which indicate a Fe–O coordination number of 6, the molecular structure of the NA-Fe nano-network was proposed, as illustrated in Fig. 2h and detailed in Table S4. The NA-Fe monomer composed of one molecule of nordihydroguaiaretic acid (NA), one Fe^3+^ ion, and four coordinated water molecules, forming the structure NA-Fe (FeC_18_O_4_ H_20_·4H_2_O). This structure enables the formation of octahedral complex with six electron-donating ligands. Hence, in addition to the two deprotonated hydroxyl ligands in NA, four water molecules serve as electron-donating ligands, facilitating the formation of a 6-coordinate complex with Fe^3+^ ion.

### 2.2 NA-Fe inactivates viruses via elevated lipoxidase-like activity

**Fig. 3:**
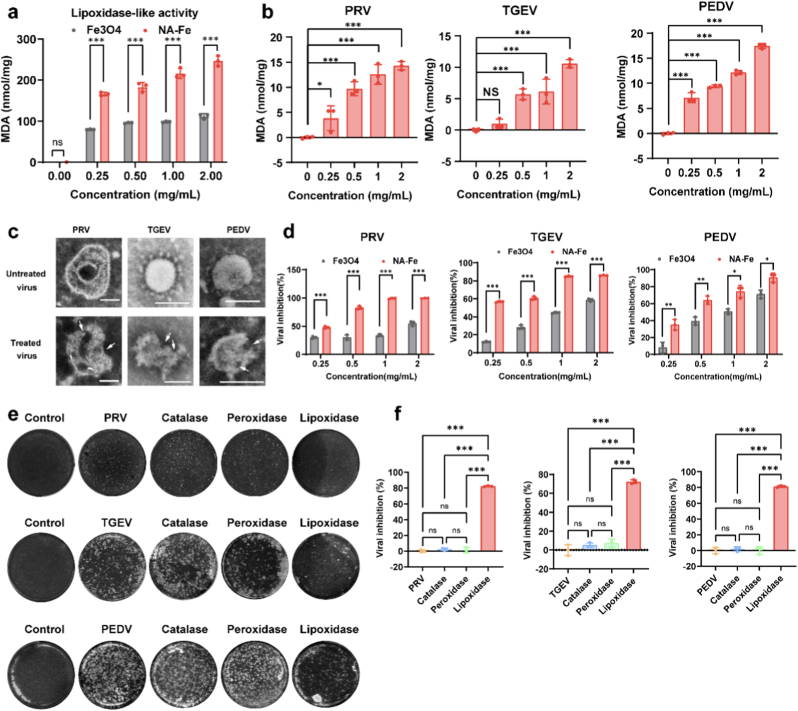
The NA-Fe inactivates the virus by lipid peroxidation. a) The level of lipid peroxidation (MDA detection) when liposomes were treated by NA-Fe and Fe_3_O_4_. b) Lipid peroxidation of PRV, TGEV, and PEDV treated by NA-Fe. c) TEM image of PRV, TGEV, and PEDV treated by NA-Fe (1 mg/mL). Scale bar: 100 nm. d) Viral plaque formation represented as a viral inhibition after treatment with different concentration of NA-Fe and Fe_3_O_4_. e-f) Bright-field image of viral plaque and viral plaque formation represented as a viral inhibition after treatment with different concentration of lipoxidase (Aladdin, L329508), or peroxidase (HRP, Solarbio, P8020), or catalase (CAT, Aladdin, C100456). All experiments were repeated in triplicate with a representative image shown. Results are presented as mean ± standard deviation (n = 3 biological replicates). Statistical significance is assessed by unpaired Student's two-sided *t*-test to the control group. ∗P < 0.05. ∗∗P < 0.01. ∗∗∗P < 0.001.

Researches have shown that the antiviral activity of nanozymes is closely related to their lipoxidase-like activity [9,10,13]. Therefore, the lipoxidase-like activity of NA-Fe was investigated by incubating it with liposomes and quantifying the amount of malondialdehyde (MDA) produced from lipid peroxidation. As shown in Fig. 3a, NA-Fe induced strong lipid peroxidation in a dose-dependent manner. Notably, the MDA levels increased by up to 2 fold compared to Fe_3_O_4_, a typical iron-based nanozymes previously demonstrated to inactivate viruses through lipid peroxidation [13]. Next, we evaluated whether NA-Fe could induce lipid peroxidation of the viral envelope by incubating it with PRV (a DNA virus) and PEDV and TGEV (RNA viruses) for 1 h at neutral pH. A high level of MDA was detected in all three viruses, and the MDA production exhibited a dose-dependent relationship (Fig. 3b). In addition, the morphology of the three viruses after NA-Fe treatment was investigated by TEM. The viral envelopes of all three viruses treated with NA-Fe were considerably damaged, as shown in Fig. 3c. Antiviral effects of NA-Fe were further evaluated by the plaque assay. The results in Fig. 3d and Figs. S5–S7 showed NA-Fe can inhibit over 90 % viral activity at the concentration of 2 mg mL^−1^ across all three viruses, significantly higher than that of Fe_3_O_4_, which exhibited an inhibition rate of around 60 %.

To confirmed that the observed viral damage was caused by the lipid peroxidation rather than other enzyme-like activities (catalase or peroxidase), the antiviral effects on all three viruses were evaluated by incubating them with natural lipoxidase, horseradish peroxidase (HRP), and catalase. The results in Fig. 3e and f demonstrated the natural lipoxidase, but not HRP or catalase was able to inhibit viral activity, implying that NA-Fe, through its lipoxidase-like activity, catalyzes the lipid peroxidation of viruses. Metal ions and polyphenolic compounds have been reported to possess antiviral activity. To investigate whether the antiviral effect of NA-Fe is attributed to the release of Fe^3+^ ions and NA (polyphenolic compounds) from NA-Fe, the concentrations of Fe^3+^ and NA released from NA-Fe were analyzed using inductively coupled plasma mass spectrometry (ICP-MS) and UV–vis spectroscopy. Analysis (Fig. S8 and Table S5) revealed that less than 0.5 % of Fe^3+^ ions (<2 μg/mL) and no polyphenolic compounds was detected over a 10-day period, further demonstrating the stability of NA-Fe nanoparticles. Subsequently, the relative concentrations of Fe^3+^ ions was assessed for their antiviral activity. Results (Fig. S9) indicated no significant antiviral activity in the Fe^3+^ ion group, possibly due to the extremely low concentrations of Fe^3+^ ions. Together, these results indicate that NA-Fe, by inducing lipid peroxidation of viral envelopes, exhibits strong antiviral effects.

### 2.3 Mechanistic insights into the lipoxidase-like activity of NA-Fe

**Fig. 4:**
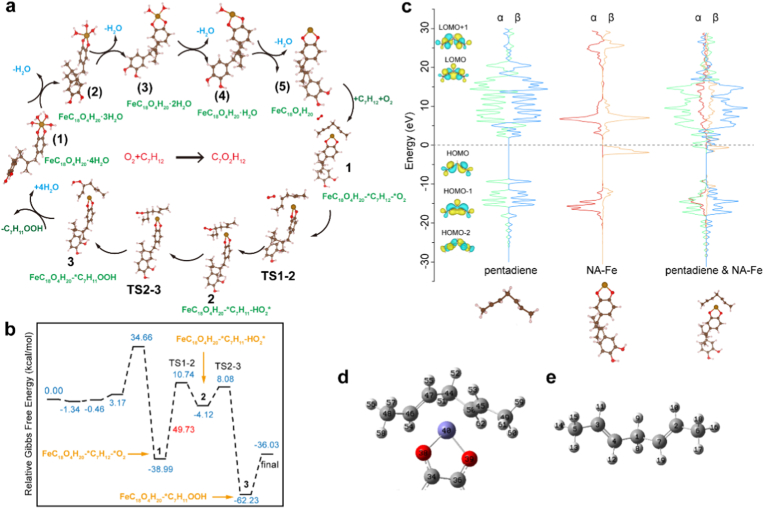
Molecular mechanism of lipoxidase-like enzymatic activity. a) The proposed reaction pathway of lipoxidase-like activity, involving the dioxygenation of a substrate containing acis,cis-1,4-pentadienemoiety on NA-Fe model. b) Gibbs free energy profile for key intermediate and transition states in the lipoxidase -like catalytic cycle. c) Electronic structure analysis of projected electronic densities of states (pDOS) of a cis,cis-1,4-pentadiene moiety, NA-Fe structure, and their interaction configuration. d) The ADCH charge of the NA-Fe after adsorbing the substrate. e) The optimized structure of acis,cis-1,4-pentadienemoiety.

The catalytic mechanism of NA-Fe was studied by density functional theory (DFT) calculation. Based on the experimental results, NA-Fe (FeC_18_O_4_ H_20_·4H_2_O) model was selected as the active agent and a cis,cis-1,4-pentadiene moiety, an important component of the viral envelop [9,11], was employed as the substrate of the lipoxidase-like catalytic activity. Based on the catalytic cycle corresponding to the dioxygenation of a substrate containing a cis,cis-1,4-pentadiene moiety by natural lipoxidase enzymes [28], a three steps mechanism was constructed to describe thelipoxidase-like activity of NA-Fe nanozyme as shown in Fig. 4a. And the step mechanism can be described by the following Eqs. (1–4), (5), (6), (7), (8)) for the lipoxidase-like activity of NA-Fe.

(1–4) FeC_18_O_4_H_20_·4H_2_O →FeC_18_O_4_H_20_ + 4H_2_O

(5) FeC_18_O_4_H_20_ + O_2_+ C_7_H_12_ →FeC_18_O_4_H_20_-∗C_7_H_12_ -∗O_2_

(6) FeC_18_O_4_H_20_ + ∗C_7_H_12_+∗O_2_ → FeC_18_O_4_H_20_-∗C_7_H_11_-HO_2_˙

(7) FeC_18_O_4_H_20_-∗C_7_H_11_-HO_2_˙ → FeC_18_O_4_H_20_-∗C_7_H_11_OOH

(8) FeC_18_O_4_H_20_-∗C_7_H_11_OOH +4H_2_O→ FeC_18_O_4_H_20_·4H_2_O + C_7_H_11_OOH

As depicted in Fig. 4a, DFT calculations suggest that the whole catalytic cycle contains eight stable states and two transition states (TS1-2, TS2-3). The dominant elementary reactions (Eqs. (5), (6), (7)), correspond to three steps) happened after FeC_18_O_4_H_20_·4H_2_O dehydration [21,29], as presented in Fig. 4a: Following the removal of bound water (step (1)–(5)), corresponding to Eqs. (1)–(4), C_7_H_12_ and O_2_ are adsorbed onto the catalyst surface (step (5)-1) corresponding to Eq. (5). Hydrogen abstraction at C-3 and radical re-arrangement to C-5 of cis, cis-1,4-pentadiene, and reacting with molecular oxygen to form a peroxy radical (FeC_18_O_4_H_20_-∗C_7_H_11_-HO_2_˙) described by Eq. (6), (corresponding to state 1 to state 2, and through the transition state TS1-2.). Peroxy anion formation and hydroperoxide generated by protonating process described by Eq. (7) (corresponding to state 2 to state 3, and through the transition state TS2-3.), leading to the formation of the product C_7_H_11_OOH. The product C_7_H_11_OOH then desorbs from the catalyst surface, restoring the Fe center of the catalyst to a 2-coordinated state. This 2-coordinated Fe species subsequently binds with four water molecules, regenerating the original 4H_2_O configuration and completing the catalytic cycle (Eq. (8)). As shown in the Gibbs free energy barrier diagram (Fig. 4b), the highest energy barrier in the entire pathway is 49.73 kcal/mol, which corresponds to the step involving the formation of the peroxy radical (FeC_18_O_4_H_20_-∗C_7_H_11_-HO_2_˙). Therefore, this step is identified as the rate-determining step of the overall reaction. Throughout the proposed reaction pathway, the change of corresponding Gibbs free energy is −36.03 kcal/mol, which indicates the catalytic cycle is feasible and easy to occur.

To elucidate the bonding nature of the species involved in the mechanism, we performed densities of states (DOS) calculations on the cis,cis-1,4-pentadiene moiety, NA-Fe, and their interaction complex (Fig. 4c). The adsorption of the substrate onto the Fe center occurs through interactions between the Fe *d*-orbitals and the carbon *p*-orbitals of the substrate. After adsorption, both the *d*-α and *d*-β electrons of Fe shift to higher energy levels compared to their pre-adsorption states, suggesting an increase in *d*-electron density on the Fe atom. For the substrate, the *p*-α electrons shift to higher energy levels, while the *p*-β electrons shift to lower levels. These opposing shifts make it challenging to conclusively determine the direction of electron transfer based solely on orbital energy levels. To clarify this, we calculated the atomic dipole corrected Hirshfeld (ADCH) charge of the substrate after adsorption (Table S6). The result shows a charge of +0.531, indicating net electron transfer from the substrate to the Fe catalyst. Further analysis of the C=C bonds before and after adsorption reveals evidence of bond activation (Fig. 4d and e and Table S7). Specifically, the left C=C bond length increases, and the corresponding bond order decreases from 1.10617 to 1.05911, indicating a weakening of the bond. Similarly, the right C=C bond elongates, with the bond order decreasing from 1.10750 to 1.02561. These changes confirm that both C=C bonds are activated upon adsorption. Researches demonstrated that free radicals and their generators have a minimal effect on antiviral activity and viral envelope disruption [25,30]. Unlike typical radical generators, NA-Fe does not generate free radicals per se, but rather catalyzes the oxidation of double bonds through specific interactions with the iron center, thereby facilitating virus inactivation via targeted lipid peroxidation.

### 2.4 NA-Fe reduced the virulence of virus and demonstrated a significant therapeutic effect during early intervention

**Fig. 5:**
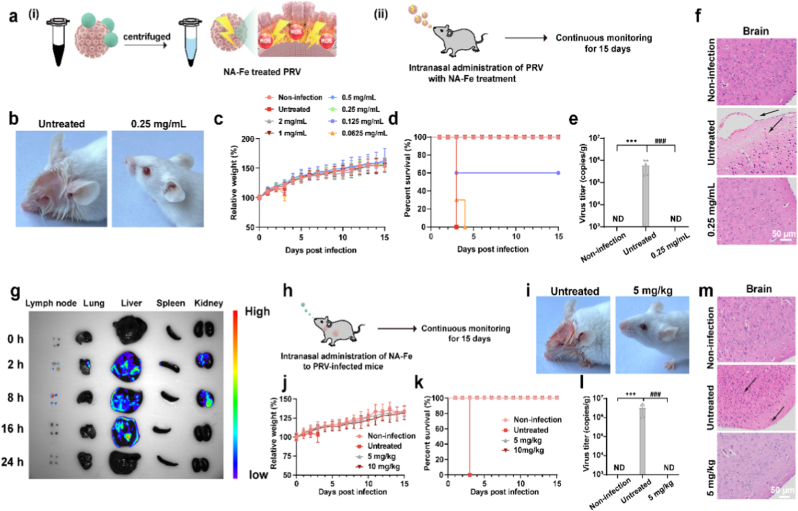
NA-Fe impaired the pathogenicity of PRV *in vivo*. a) Schematic diagram of the virus inoculated with mice after it has been incubated with the NA-Fe. b) Representative photographs of mice on day 3 post infection without and with NA-Fe treatment (0.25 mg/mL). c-d) Body weight and survival rate of mice was monitored for 15 consecutive days (n = 10). e) Copies of PRV genome per gram brain of mice at 3 dpi. f) Effect of NA-Fe (0.25 mg/mL) on the microstructures of brains of mice at 3 dpi (n = 3). The application of NA-Fe in prevention of PRV infection in the early stage. g) Fluorescence imaging of the isolated organs from mice at different time points after intranasal administration of NA-Fe. h) The Schematic diagram illustrates the infection process and protocol of NA-Fe treatment. i) Representative photographs of mice on day 3 postinfection without and with NA-Fe treatment (5 mg/kg). j-k) Body weight and survival rate of mice was monitored for 15 consecutive days (n = 10). l) Copies of PRV genome per gram brain of mice at 3 dpi. m) Effect of NA-Fe (5 mg/kg) on the microstructures of brains of mice at 3 dpi (n = 3). All experiments were repeated in triplicate with a representative image shown. Results are presented as means ± standard deviation (n = 3 biological replicates). ∗∗∗ indicates p < 0.001 compared to the mock group. ^###^ indicates p < 0.001 compared to the untreated group.

Given that PRV induces disease in mice, we utilized both the virus and a murine model to investigate the viral inactivation efficacy of NA-Fe. BALB/c mice were intranasally infected with either untreated PRV or NA-Fe-treated PRV. Mice infected with untreated PRV exhibited significant body weight loss and a 0 % survival rate within 3 days post-infection (dpi). In contrast, mice infected with NA-Fe-treated PRV (0.0625 mg mL^−1^) all died on the fourth day. Notably, the survival rate in the 0.125 mg mL^−1^ group increased to 60 %, while mice in groups treated with concentrations above 0.25 mg mL^−1^ maintained their body weight and achieved a 100 % survival rate (Fig. 5a–d). In addition, the level of viral gene expression in the brains of mice in the NA-Fe-treated PRV group was significantly reduced compared to that in mice infected with untreated PRV (Fig. 5e). Histopathological analysis of brain tissues following hematoxylin and eosin (HE) staining revealed neuronal degeneration and hemorrhage in the brains of mice infected with untreated PRV, whereas these pathological changes were not observed in the non-infection group and NA-Fe-treated PRV group at a concentration of 0.25 mg mL^−1^(Fig. 5f). Similarly, in the lungs of mice infected with intact PRV, the alveolar structure was disrupted, accompanied by inflammatory cell infiltration, hemorrhage, and congestion. Conversely, no apparent pathological alterations were observed in the lungs of mice in the NA-Fe-treated PRV group. Relevant pathological changes were also observed in the heart, liver, and kidney of untreated PRV-infected mice but not in NA-Fe-treated PRV group (Fig. S10). These results clearly demonstrated that NA-Fe can significantly reduce the virulence of virus.

Next, biosafety assays both *in vitro* and *in vivo* were conducted prior to evaluating the therapeutic effects of NA-Fe. *In vitro,* cytotoxicity assays (Fig. S9) demonstrated that both PK15 cells (host of PRV and TGEV) and Vero cells (host of PEDV) maintained nearly 100 % viability even the concentration of NA-Fe up to 1000ug mL^−1^, suggesting the good biocompatibility of NA-Fe. *In vivo*, intranasal administration of NA-Fe at a dose of 10 or 5 mg kg^−1^ did not significantly affect body weight, and the survival rate of the mice remained at 100 % with traced for 15 days (Fig. S11). Histological analysis revealed no pathological changes in the nasal cavity or spleen tissues of treated mice; their structural integrity remained intact. Similarly, no abnormalities were observed in the lungs, heart, liver, or kidneys (Fig. S12), further confirming the biosafety of NA-Fe. To investigate the biodistribution and metabolism of NA-Fe, fluorescence imaging was performed following nasal administration at a concentration of 10 mg kg^−1^. The results showed that NA-Fe accumulated transiently in the lymph nodes, lungs, liver, spleen, and kidneys, but was efficiently metabolized and cleared from the body within 24 h (Fig. 5g). These results suggest that NA-Fe exhibits good biocompatibility.

To assess the antiviral efficacy of NA-Fe *in vitro*, host cells (PK15 and Vero) were infected with PRV, PEDV, or TGEV for 1 h, followed by treatment with varying concentrations of NA-Fe. Viral infectivity was quantified using a plaque assay, and the results were expressed as half-maximal effective concentration (EC_50_) in Fig. S13. NA-Fe treatment led to a significant reduction in viral titers across all three viruses. Specifically, the EC_50_ values of NA-Fe were 172.4 μg mL^−1^ for PRV, 169.6 μg mL^−1^ for TGEV, and 5.5 μg mL^−1^ for PEDV, indicating potent and broad-spectrum antiviral activity. After testing the half-maximal cytotoxic concentration (CC_50_) of NA-Fe, the Selection Index ($\text{SI}=\frac{{\text{CC}}_{50}}{{\text{EC}}_{50}}$) can be calculated, serving as a standard for evaluating the antiviral effect. Notably, according the cytotoxicity results (Fig. S14), the SI can be calculated as listed in Table S8. The results demonstrated that NA-Fe exhibits low cytotoxicity and high antiviral efficacy, supporting its potential as a safe and effective antiviral agent *in vitro*.

*In vivo* assay, mice were intranasally infected with PRV (10^3^ TCID_50_), followed by intranasal administration of NA-Fe at doses of 5 mg kg^−1^ or 10 mg kg^−1^ at 6 h post infection (hpi). Mice treated with NA-Fe, both 5 mg kg^−1^ and 10 mg kg^−1^, maintained their body weight and showed 100 % survival rate over a 15-day observation period, whereas all mice were died in the group of non-treatment with NA-Fe at 3 dpi (Fig. 5h–k). Viral gene levels in brain of the therapeutic group (NA-Fe treatment) were significantly lower than those in the infection group (Fig. 5l). Histopathological analysis of brain tissues following hematoxylin and eosin (HE) staining revealed neuronal degeneration and hemorrhage in the brains of mice infected PRV, whereas these pathological changes were not observed in the group treated with NA-Fe (Fig. 5m). Similar protective effects were also observed in other organs, including the liver, heart, spleen, lungs, and kidneys (Fig. S15). Collectively, these findings demonstrate that NA-Fe confers significant therapeutic efficacy when administered during the early stages of PRV infection.

### 2.5 NA-Fe exhibits antiviral activity by activating the host innate immune system

Inspired by the significant antiviral therapeutic effects of NA-Fe during early intervention, we further investigated the mechanism of interaction between NA-Fe and the viral host system. Viral plaque test was applied to determine whether NA-Fe inhibits replication of intracellular viruses, corresponding experiments were designed accordingly (Fig. S16). The virus first infects the cells for 1 h, after which NA-Fe is applied for 24 h. The results, expressed through plaque formation and viral inhibition in Fig. S17, showed that NA-Fe can suppress the intracellular replication of all three viruses.

**Fig. 6:**
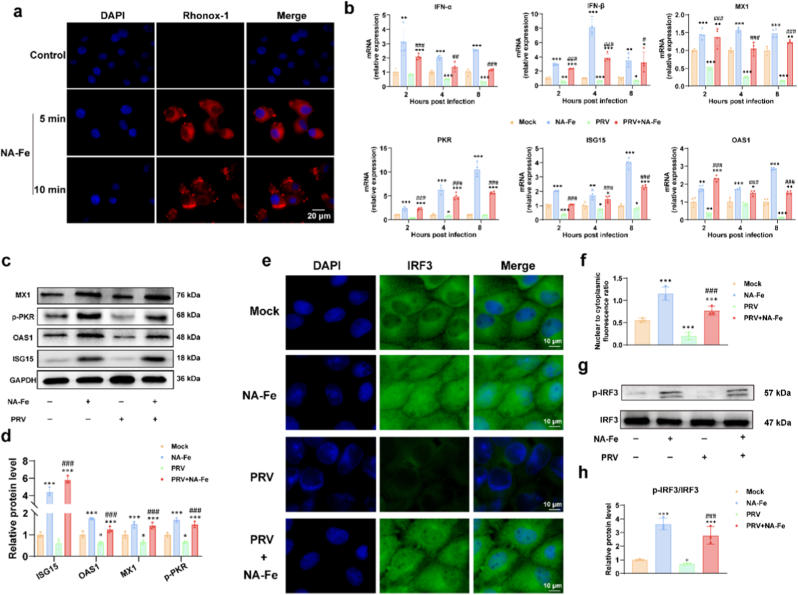
a) Rapid cellular internalization of NA-Fe (NA-Fe marked by RhoNox-1, red. Scale bar: 20 μm, 400× magnification). b) Type I IFNs and ISGs mRNA expression levels in PK-15 cells induced by NA-Fe. β-actin was used to normalize the gene expression. PK-15 cDNA samples were used for real-time RT-qPCR analysis of the expression levels of IFN-α, IFN-β, MX1, OAS1, ISG15, and p-PKR. Data are shown as the mean values ± standard deviations from three replications. c) Western blot images of MX1, OAS1, ISG15, PKR at 8 hpi. d) The relative protein level of ISGs quantified by ImageJ software. e) Immunofluorescence assay (1000× magnification) was carried out to observe the nuclear translocation of IRF3 at 8 hpi. Scale bar: 10 μm. Blue represents the nucleus and green represents the IRF3. The field of view is random. f) The nuclear to cytoplasmic fluorescence ratio ± standard deviations of IRF3. g) Expression of phosphorylation IRF3 at 8 h performed by western blot (n = 3). h) Relative protein level of p-IRF3/IRF3 quantified by ImageJ. All experiments were repeated in triplicate with a representative image shown. Results are presented as means ± standard deviation (n = 3 biological replicates). ∗∗∗ indicates p < 0.001 compared to the mock group. ^###^ indicates p < 0.001 compared to the PRV group. (For interpretation of the references to color in this figure legend, the reader is referred to the Web version of this article.)

To investigate the interaction between NA-Fe and host cells, PK15 cells were incubated with NA-Fe, and cellular uptake was evaluated. As shown in Fig. 6a, NA-Fe successfully entered the cells within 5 min of incubation, indicating rapid cellular internalization. Given that the replication stage is known to be closely associated with the host's innate immune response [6,[31], [32], [33], [34]], we next investigated whether NA-Fe modulates the expression of type I interferons (IFNs). Time-dependent expression of IFNs following NA-Fe treatment was determined by using reverse transcription-quantitative polymerase chain reaction (RT-qPCR). As shown in Fig. 6b, NA-Fe significantly upregulated the mRNA expression levels of type I IFNs (IFN-α and IFN-β) in PK15 cells, both in the presence and absence of PRV infection. Innate immunity serves as the first line of defense and plays a crucial role in the host's early response to viral infections. The results indicated that at the early stage of PRV infection (2,4,8 hpi), NA-Fe could stimulate the innate immune response. Elevated IFN levels are known to activate interferon-stimulated genes (ISGs), which play key roles in antiviral and inflammatory responses. Among these, ISG15, OAS1, MX1, and phosphorylated PKR (p-PKR) are recognized for their positive regulation of antiviral pathways, including the activation of phosphorylated signal transducer and activator of transcription proteins and retinoic acid-inducible gene I (RIG-I)-like receptors, which suppress viral replication [[35], [36], [37], [38]]. Therefore, we analyzed the mRNA and protein expression levels of ISG15, OAS1, MX1, and p-PKR using RT-qPCR and Western blotting. As shown in Fig. 6b–d, NA-Fe treatment led to significant upregulation of both mRNA and protein levels of these ISGs in PK15 cells, regardless of PRV infection status. Additionally, the nuclear expression of phosphorylated interferon regulatory factor 3 (p-IRF3), a key early regulator of type I IFN production downstream of intracellular viral sensing, was also enhanced following NA-Fe treatment (Fig. 6e–h). Collectively, these findings indicate that NA-Fe activates innate immune responses by promoting IRF3 phosphorylation and nuclear accumulation of p-IRF3, and by upregulating the expression of key ISGs at both the mRNA and protein levels.

### 2.6 NA-Fe-loaded farm fence and air filter provided excellent protection against viruses

**Fig. 7:**
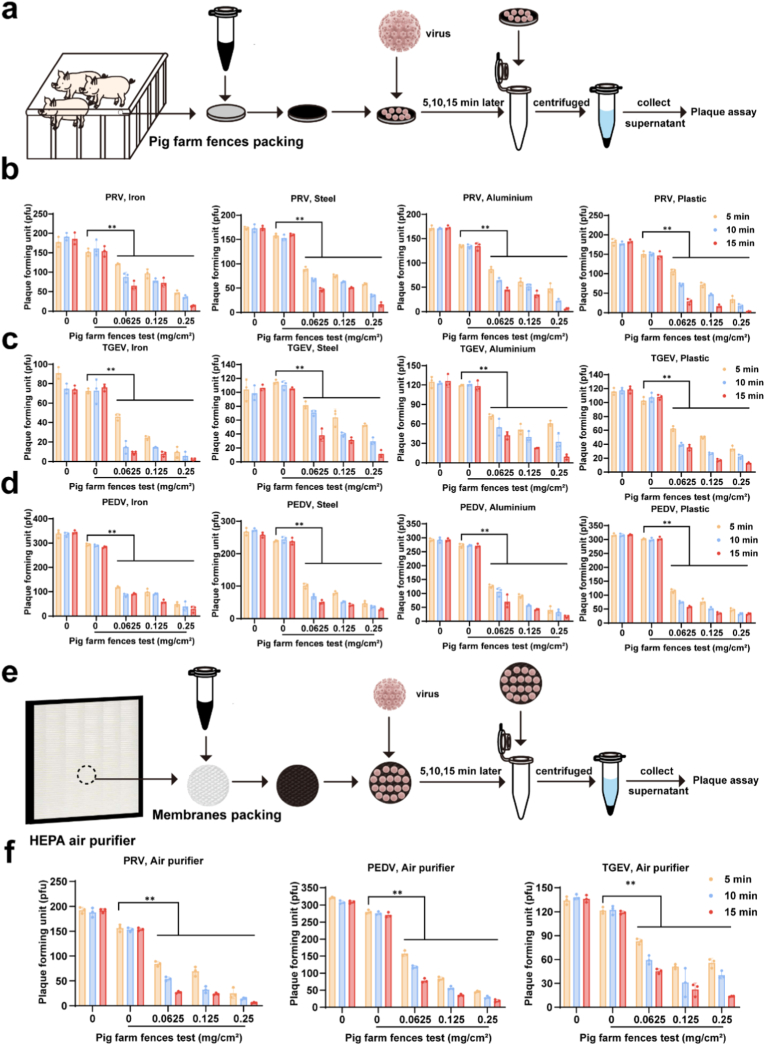
The applications of NA-Fe against PRV, TGEV, and PEDV. a) Flow diagram of the application of NA-Fe to pig farm fences. b-d) Viral plaque forming unit after treatment with different concentration of NA-Fe coated on the different materials that make up the fence of the pig farm. e). Flow diagram of the application of NA-Fe to HEPA air purifier. f) Viral plaque forming unit after treatment with different concentration of NA-Fe coated on fiberglass membranes. All experiments were repeated in triplicate with a representative image shown. Results are presented as means ± standard deviation (n = 3 biological replicates). ∗∗∗ indicates p < 0.001 compared to the mock group.

Animal health is closely linked to human and environmental well-being, with the breeding environment of animals playing a direct and significant role in determining their overall health status. Here, we evaluate the antiviral effectiveness of NA-Fe when coated onto animal breeding fences and air filters. Since fences (Fig. 7a) are typically constructed from materials such as steel, iron, aluminum, or plastic, we applied NA-Fe onto the surfaces of these materials through a coating process with variety concentration (0.0625, 0.125, and 0.25 mg/cm^2^). SEM imaging (Fig. S18) reveals uniformly distributed particles adhered to the surfaces of both the fence materials and the air filter. Then, three viruses incubated to the surfaces of fence with different time (5, 10, 15 min), the antiviral efficiency determined by plaque assay. The result show, virus titers of PEDV, TGEV, and PRV were significantly reduced when the fence materials coated with NA-Fe. In particular, at a high concentration of 0.25 mg/cm^2^, PRV (Fig. 7b), TGEV (Fig. 7c), and PEDV (Fig. 7d) were almost completely inactivated within 15 min. In addition, similar phenomena was observed on the air filters (Fig. 7e and f), in which all three viruses were almost completely inactivated within 15 min with the concentration of 0.25 mg/cm^2^. The potent antiviral efficiency stops the virus transmission to animal and other objects, which is quite important to the healthy animal breeding. Although chemical disinfectants and ultraviolet (UV) equipment possess antiviral properties, their effectiveness is limited throughout the entire process due to the volatility of chemical agents and the need for specialized equipment [1,39,40]. The antiviral activity of NA-Fe on different surfaces has been demonstrated, which enabled NA-Fe to inactivate viruses at different breeding facility environment.

## 3 Discussion

This study proposed a forward-looking strategy for using NA-Fe to fight against enveloped viruses, which exhibits the following characteristics: 1) NA-Fe has high lipoxidase-like activity to oxidize the viral envelope. 2) NA-Fe has an inactivating effect on a variety of enveloped viruses, including RNA viruses: PEDV and TGEV; DNA viruses: PRV. 3) NA-Fe inactivates viruses by catalyzing the oxidation of polyunsaturated fatty acids (C=C bonds) in the viral envelope. 4) NA-Fe can enter cells in a short time, inhibit the replication of intracellular viruses by activating the host's natural immunity, and has a therapeutic effect on mice initially infected with PRV. 5) NA-Fe can be used as a coating on farm fences and air filters to effectively inhibit the spread of viruses. In general, these characteristics provide a comprehensive antiviral strategy for enveloped viruses, which is expected to be applied in the biomedical field.

Nanonetworks formed through the chelation of natural polyphenols with Fe^3+^ ions have demonstrated high nanozyme activity [41], along with notable antibacterial [20] and antitumor [21] properties. However, their antiviral potential and underlying mechanisms of action remain largely unexplored. Moreover, previously reported nanonetworks often suffer from several limitations, including large particle sizes, irregular morphology, poor cellular uptake, and unclear metabolic pathways *in vivo*. In this study, we report for the first time the successful synthesis of NA-Fe nanozymes through the chelation of NA with Fe^3+^ ions. The resulting NA-Fe nanozymes exhibit uniform morphology, small particle size, high catalytic activity, excellent biocompatibility, rapid *in vivo* metabolism, and strong structural stability. Notably, NA-Fe has strong lipoxidase-like activity and can oxidize viral envelopes with polyunsaturated fatty acids. DFT calculations and DOS analysis further revealed that upon adsorption of unsaturated fatty acids, NA-Fe is capable of capturing electrons from the C=C bonds. This electron transfer activates the double bonds, thereby accelerating the oxidation process and contributing to the nanozyme's antiviral mechanism.

Our results demonstrate that NA-Fe can inactivate a variety of enveloped viruses including DNA viruses PRV, RNA viruses (PEDV and TEGV). These findings suggest that NA-Fe exhibits no apparent virus specificity and may possess broad-spectrum antiviral activity against enveloped viruses. NA-Fe exhibits strong antiviral effects *in vitro* and shows great potential for application in agricultural and farming environments. In contrast to conventional disinfectants like alcohol, hydrogen peroxide, and ultraviolet light, which have short durations of action, high volatility, or environmental toxicity, NA-Fe demonstrates stable physicochemical properties, indicating significant potential as a substitute for disinfectants. A limitation of the current study is the lack of direct comparison between NA-Fe and other nanozymes or conventional antiviral agents. Therefore, in future work, we plan to systematically compare the antiviral efficacy of NA-Fe with other nanozyme-based materials as well as with common disinfection methods, such as alcohol, hydrogen peroxide, and ultraviolet (UV) treatment, in order to better evaluate its practical applicability and advantages. In addition to its extracellular antiviral effects, NA-Fe's small particle size facilitates rapid cellular uptake, allowing it to activate the host's innate immune response and inhibit intracellular viral replication. *In vivo* studies further demonstrate its therapeutic efficacy in mice during the early stages of viral infection. These results highlight the dual functionality of NA-Fe in targeting both extracellular and intracellular virus. Moreover, the ability of NA-Fe to stimulate innate immune pathways suggests a potential advantage against rapidly mutating enveloped viruses, where conventional therapies may be less effective. Existing researches show that nanomaterials have significant antiviral potential *in vitro* [1,5,6,40,[42], [43], [44]], while relatively few have been evaluated for their efficacy *in vivo*. Moreover, concerns regarding the cytotoxicity and biocompatibility of these nanoparticles have raised critical issues related to their environmental safety and potential risks to human and animal health. Unlike many antiviral nanomaterials with unclear biosafety profiles and undefined metabolic pathways, NA-Fe exhibits high biosafety both *in vitro* and *in vivo*, along with a well-defined metabolic pathway, thereby supporting its safe and effective application in biosecurity and infectious disease prevention. In the current study, we evaluated the biosafety of NA-Fe *in vivo* over a 15-day period. Long-term biosafety and the potential accumulation of NA-Fe nanoparticles in tissues will be investigated in future studies to further confirm the safety profile of NA-Fe. Collectively, these attributes position NA-Fe as a promising broad-spectrum antiviral agent, particularly suited for combating emerging and re-emerging viral threats.

In conclusion, our results demonstrate that NA-Fe exhibits excellent performance against both extracellular and intracellular enveloped viruses. These characteristics endow NA-Fe with good adaptability to combat pandemics caused by enveloped viruses. It may be a suitable choice for the prevention and treatment of epidemics caused by enveloped viruses in the future.

## 4 Experimental section

*Synthesis of Phenolic-Fe Networks:* Nordihydroguaifenacic acid, Proanthocyanidins, Ellagic acid and Epigallocatechin gallate was purchased from Aladdin Chemistry Co. Ltd. (Shanghai, China). NaAc (AR) were obtained from the Chron Chemicals Co. Ltd. (Cheng Du, China). DMSO were obtained from Lanjieke Technology Co. Ltd. (Beijing, China). All reagents were directly used without further purification. Briefly, an aliquot of NA in DMSO (40 μL, 30 mM) was added to sodium acetate buffer (3.94 mL, 56 mM, pH 4.5) under stirring for over 1 min. Then Iron chloride hexahydrate in ultrapure water (40 μL, 10 mM) was added dropwise to the above reaction mixture under stirring for 5 min. NA-Fe was obtained after being stirred at room temperature for 1 h. The other phenolic-Fe was prepared similarly.

*Characterization of Samples:* The morphology of the samples was measured with a transmission electron microscope (JEM-2100 plus, JEOL, Japan). The zeta potential was measured by dynamic light scattering (DLS) using a Malvern Zeta sizer instrument (Malvern nano s90, Malvern, UK). Thermogravimetric analysis (SDT650, TA instruments, US) was used to check the thermal stability under a nitrogen atmosphere from RT to 1000 °C. A Fourier transform infrared spectrometer (Spectrum Two FT-IR Spectrometer, PerkinElmer, USA) was used to analyze possible functional groups existing in the samples. The UV–vis absorption of all samples was measured on a UV-6100 spectrophotometer (MAPADA, China). X-ray diffraction (XRD) analyses were conducted on a Malvern Panalytical X-ray diffractometer with CuKα radiation (λ = 1.5406 Å) to confirm the crystalline structures of NA-Fe. For X-ray photoelectron spectroscopy (XPS) analysis, the freeze-dried NA-Fe powder was finely ground using an agate mortar, and measurements were performed with an EscaLab 250Xi spectrometer.

*XAFS data processing:* The extended X-ray absorption fine structure (EXAFS) measurements were carried out on the sample BL11 s hatch X-ray absorption beamline of SAGA Light Source. This beamline adopted a Si (1 1 1) monochromator X-ray absorption spectroscopy. The end-station is equipped with an ionization chamber positioned in front of the sample, along with two ionization chambers and a seven-element SDD detector located behind the sample, enabling both transmission and fluorescence mode X-ray absorption spectroscopy. The energy resolution of the station ranges from 10^−4^ to 10^−3^ ΔE/E, with a photon flux ranging from 2 × 10^9^ to 1 × 10^8^ photons/sec, corresponding to X-ray energies in the range of 2.1 keV–14 keV. The obtained XAFS data was processed in Athena (version 0.9.26) for background, pre-edge line and post-edge line calibrations. Then Fourier transformed fitting was carried out in Artemis (version 0.9.26). The k^2^ weighting, *k*-range of 3–14 Å^−1^ and R range of 1–∼3 Å were used for the fitting of Fe foil; *k*-range of 3–11.5 Å^−1^ and R range of 1–∼3 Å were used for the fitting of samples. The four parameters, coordination number, bond length, Debye-Waller factor and E_0_ shift (CN, R, ΔE_0_) were fitted without anyone was fixed, the σ^2^ was set. For Wavelet Transform analysis, the χ(k) exported from Athena was imported into the Hama Fortran code. The parameters were listed as follow: R range, 1–4 Å, k range, 0–12 Å^−1^ for samples; k weight, 2; and Morlet function with κ = 10, σ = 1 was used as the mother wavelet to provide the overall distribution.

*Cells and Viruses:* Porcine kidney (PK-15) cells (RRID: CVCL_2160, contamination-free) and African green monkey kidney (Vero) cells (RRID:CVCL_U963, contamination-free) were purchased from the China Center for Type Culture Collection (CCTCC). Cells were preserved at 37 °C in a 5 % CO_2_ atmosphere as adherent culture in Dulbecco's modified Eagle's medium (DMEM/High glucose, Gibco) mixed with 10 % newborn calf serum (NBCS, Viva cell). When grown to 80–90 % confluence, the cells were washed with phosphate buffer saline (PBS) and collected from the culture vessel surface by adding 0.25 % trypsin. PRV (Rong A strain, purchased from China Veterinary Culture Collection Center) was propagated in PK-15 cells cultured in maintenance mediums (DMEM containing 2 % NBCS). TGEV (SC2021, purchased from China Animal Husbandry Industry Co. Ltd) was propagated in PK-15 cells cultured in maintenance mediums DMEM containing 2 % NBCS. PEDV (ZJ08, purchased from China Animal Husbandry Industry Co. Ltd) was propagated in Vero cells cultured in maintenance mediums (DMEM containing 5 μg/mL trypsin).

DFT Calculation of Lipoxidase-like activity: All DFT calculations were carried out using ORCA (version: 5.0.3) [[45], [46], [47]]. The density of states, LUMO and HOMO orbitals, Atomic dipole corrected Hirshfeld atomic charge (ADCH) [48], and Intrinsic bond strength index (IBSI) [49,50] were analyzed with MULTIWFN [51,52]; and the results were visualized with VMD software [53]. In the DFT calculations, geometry optimization was conducted at the B3LYP/6-31G(d,p) level of theory, which were dispersion corrected by D3BJ [[54], [55], [56]]. The solvent effect was evaluated by the CPCM solvent model. Vibrational frequency analysis was carried out to identify the nature of each stationary points as a minimum or a transition state, and to acquire the Gibbs free energy correction. Accurate energy was obtained by performing the single point calculations for the optimized geometries at the ωB97X-V/def2-TZVP level of theory [57]. The RIJCOSX approximation was applied with the def2/J auxiliary basis set [58,59]. The Gibbs free energies were included in the Gibbs energy correction of unscaled vibrational analysis at the B3LYP/6-31G(d,p) (D3BJ) level of theory.

Peroxidase-like Activity and Kinetic Assay: To study the kinetics of peroxidase mimicking activity of NA-Fe, NA-Fe in water (300 μL, 0.1 mg/mL) and TMB in DMSO (30 μL, 20 mM) were mixed in acetate buffer (2.64 μL, 200 mM, pH 4.5). Then H_2_O_2_ in water (30 μL) was added to a final H_2_O_2_ concentration ranging from 0 to 100 mM. The mixture was incubated at 37 °C for 10 min before the absorbance at 652 nm was measured on a Thermo Scientific Varioskan Flash multimode microplate reader. In a parallel study, NA-Fe in water (300 μL, 0.1 mg/mL) and H_2_O_2_ in ultrapure water (30 μL, 100 mM) were mixed in acetate buffer (2.64 μL, 200 mM, pH 4.5). Then TMB in water (30 μL) was added to a final TMB concentration ranging from 0 to 8 mM. The mixture was incubated at 37 °C for 10 min before the absorbance at 652 nm was measured on a Thermo Scientific Varioskan Flash multimode microplate reader. The apparent kinetic parameters were calculated based on the function v = Vmax × [S]/(Km + [S]), where v is the initial velocity, Vmax is the maximal reaction velocity, [S] is the concentration of substrate, and Km is the Michaelis constant.

*Determination of lipid peroxidation:* MDA levels, a reliable marker of lipid peroxidation, were measured using thiobarbituric acid (TBA). Different concentrations of NA-Fe and Fe_3_O_4_ were mixed with liposomes for 1 h. After centrifugation, the supernatant was collected. In addition, NA-Fe were mixed with PRV/TGEV/PEDV for 1 h. After centrifugation, the supernatant was also collected to detect the levels of lipid peroxidation using a commercial MDA detection kit according to the manufacturer's instruction (Nanjing Jiancheng Bioengineering Institute, A003-1). MDA concentrations were calculated on the basis of the absorbance of TBA reactive substances (TBARS) at 530 nm.

*Cytotoxicity Assay:* When grown to 80–90 % confluence, PK-15/Vero cells were seeded in 96-well plates, followed by incubation separately with NA-Fe at different concentrations in DMEM supplemented with 2 % NBCS for 24 and 48 h. After discarding the supernatant, each well was supplemented with DMEM containing 5 % CCK-8 reagent. After incubation for 40 min at 37 °C in the dark, the mixture in the plates was collected, followed by centrifugation at 10,000 g for 10 min, and the supernatant was collected and added to new plates. The absorbance values were measured using a microplate reader (Bio-rad, USA) at 450 nm. The 50 % cytotoxic concentration (CC_50_) was calculated as the compound concentration required for reducing cell viability by 50 %.

*The Detection of Effect concentration of 50 %*: Maintenance mediums containing virus (MOI = 0.1) were preincubated with a serial two-fold dilution of NA-Fe for 1 h at 37 °C. Then the mixtures were added to cell monolayers in 96-well plates, and the plates were incubated at 37 °C for 1h to allow the virus attachment. Thereafter, the mediums were aspirated to remove the unabsorbed virus. The cell monolayers were then washed with PBS, and maintenance mediums containing serial two-fold dilutions of samples were added to the plates. The untreated cells and uninfected cells were used as controls. All plates were incubated at 37 °C in a 5 % CO_2_ atmosphere. After 48 h, the plates were frozen-thawing three times and the supernatant was collected as the sample and stored at −80 °C refrigerator. The amount of virus in the sample was quantified by plaque assay. The 50 % effective concentration (EC_50_) was the concentration that reduced 50 % of the plaque-forming unit (pfu) relative to that of the virus control. Selection Index (SI) means the ratio of half toxic concentration (CC_50_) to half effective concentration (EC_50_).

*Plaque Assay:* Following growth to confluence in 6- or 12-well plates, cells were infected with virus samples that were either untreated, NA-Fe-treated, or treated with 2 μg/mL Fe^3+^ (FeCl_3_). Virus was diluted in maintenance medium via serial dilution before addition to wells. After 1 h of adsorption, the supernatant was removed and the cells were washed twice with PBS to get rid of the non-absorbed virus particles. After adding to each well 2 ml of plaque fluid (50 % 2 × DMEM and 50 % low melting point agarose), the plates were turned upside down and incubated at 37 °C for 2–4 days until the plaque fluid were completely solidified. Then, the cells were stained at 37 °C for 2 h after adding to each well the 1:1 mixture of 2 ml of crystal violet solution (1 %) and formaldehyde (20 %).

*Replication Assay:* When cells were cultured to 90 % confluence in 96-well plates, the cells were washed with PBS and then were infected with the virus (MOI = 0.1) at 37 °C for 1 h. Next, the virus inoculum was discarded and the cells were washed twice with PBS to remove non-adsorbed virus particles. Subsequently, the cells were incubated separately with the maintenance medium in the absence or presence of 0.50 mg mL^−1^ NA-Fe for 24 h. Then, the 96-well plates were placed in the −80 °C refrigerator and frozen and thawed three times repeatedly and the supernatant was collected as the sample, followed by plaque assay.

*Electron Microscopy:* The purified virus was incubated with NA-Fe for 1 h at 37 °C followed by dropping it on a copper grid for 5 min and then negatively staining with phosphotungstic acid (PTA, pH 7.0). After drying, grids were examined using an electron microscope (JEM-2100 plus, JEOL, Japan).

*Biosafety Evaluation of NA-Fe:* Thirty female specific pathogen-free BALB/c mice (body weight 20 ± 2 g) were commercially obtained from the Chengdu Dossy Experimental Animals Co., Ltd. (Chengdu, China) and raised in the laboratory animal center of Sichuan Agricultural University (Chengdu, China). After one week of adaptation, the mice were divided into three groups and each group contained 10 mice. Except for the mock group, the mice were intranasally treated with 5 or 10 mg/kg NA-Fe by nasal dropping administration. Body weight and survival rate were monitored for 15 days. Nasal cavitys, organs (n = 3) were collected for histopathological examination at 7 days post-infection (dpi). All animal experiments conformed to the Guide for the Care and Use of Laboratory Animals from the National Institutes of Health, and all procedures were approved by the Animal Research Committee of Sichuan Agricultural University [permission number 20230074].

*Virulence of NA-Fe-treated PRV in Mice:* Firstly, PRV was incubated with different concentrations of NA-Fe at 37 °C for 1 h. Then, the mice (n = 10) were intranasally inoculated with 1000 TCID_50_/10 μL of each treated or untreated PRV. Body weight and survival rate were monitored for 15 days. Mice brains were collected to detect viral replication levels at 3 days post-infection (dpi). Meanwhile, mice brains and organs (n = 3) were collected for histopathological examination at 3 dpi.

*In Vivo Antiviral Efficacy of NA-Fe:* Forty female specific pathogen-free BALB/c mice (body weight 20 ± 2 g) were reared normally, as previously described. Except for the mock group, the mice were infected intranasally with PRV (1000 TCID_50_). After 6 h, the mice were intranasally treated with10 μL of PBS (PRV group) and NA-Fe at a dose of 5/10 mg kg^−1^ of body weight in PBS by nasal dropping administration. Survival rates and body weight were recorded daily for 15 days. Mice brains and were collected to detect viral replication levels at 3 days post-infection (dpi). Meanwhile, mice brains and organs (n = 3) were collected for histopathological examination at 3 dpi.

*Viral load assay:* The copies of the PRV genomes were detected by FQ-PCR assay. The mice brains were collected from each group and stored in the −80 °C refrigerator, followed by liquid nitrogen homogenization. Then the total DNA of the tissue sample was extracted by using an Easy Genomic DNA Kit (Transgen, EE101) according to the manufacturer's instructions and analyzed using SsoAdvancedTM Universal Probes Supermix (Bio-Rad, 1725281). The reaction contained in a final volume of 20 μl: SsoAdvancedTM Universal Probes Supermix (10 μl), each primer set (0.5 μl), and purified DNA (2 μl), DNase-free water (6.5 μl). The FQ-PCR was performed as one cycle of 3 min at 95 °C followed by 40 cycles of 5 s at 95 °C and 30 s at 54 °C. The gene copies were calculated according to the standard curve: lg [virus copies] = -0.3321 Cq+13.902 (R^2^ = 0.983). The gB primers used to amplify a 95-bp fragment of the glycoprotein B gene of PRV (GenBank accession no. KJ526438) and the probe are shown in Table S9.

*Histopathological examination:* During dissection, the nasal cavities, hearts, livers, spleens, lungs, kidneys, and brains of mice were taken and fixed in 4.0 % paraformaldehyde, followed by embedding in paraffin. Sections (5 μm) were cut and stained with the hematoxylin-eosin (HE) solution. Histopathological changes were observed under a pathology slide scanner (Olympus, VS120-S6-W, Japan). Three slides from different parts of each tissue type (3 mice per group) were analyzed.

*Cellular uptake assays of NA-Fe:* Firstly, NA-Fe was successfully marked by an activatable fluorescent probe (RhoNox-1) which combined with Fe irons specially. Then, PK-15 cells were collected to detect Red fluorescence intensity by Fluorescence microscopy after incubating with RhoNox-1-marked NA-Fe for 5 and 10 min. All experiments were repeated in triplicate with a representative image shown.

*Indirect Immunofluorescence Assay (IFA):* PK-15 cells were seeded on the cell climbing tablets in a 24-well plate at low densities and cultured to 70–80 % confluence at 37 °C and 5 % CO_2_. The cells were infected with the virus (MOI = 0.1) at 37 °C for 1 h. Next, the virus inoculum was discarded and the cells were washed twice with PBS to remove non-adsorbed virus particles. Subsequently, the cells were incubated separately with the maintenance medium in the absence or presence of 0.50 mg mL^−1^ NA-Fe. After 8 h, the cells were washed three times with PBS, fixed with 400 μl/well of 4 % paraformaldehyde for 15 min at room temperature (RT), and permeabilized with 400 μl/well of 0.2 % Triton-X100 for 15 min at RT. After three washes with PBS (5 min for each wash), the cells were blocked with 400 μL/well of 5 % bovine serum albumin (BSA) at RT for 1 h. Then, for the detection of nuclear factors IRF3, the cells were incubated with a primary antibody (Proteintech, 11312-1-AP). After being washed three times with PBS, the cells were incubated with CoraLite488-conjugated Goat Anti-Rabbit IgG (Proteintech, SA00013-2) in the dark at RT for 1 h. Subsequently, the cells were dyed with DAPI for 15 min in the dark, followed by three washes with PBS. Fluorescence microscopy (Zeiss, Axio Imager M2, DE) was used to obtain the fluorescence images.

*Gene expression analysis:* The PK-15 cells grown in 6-well plates were infected with or without PRV at an MOI = 1 for 1h at 37 °C. After washing with PBS, maintenance medium with or without NA-Fe (0.5 mg/mL) was added to the cultures after removal of the virus inoculums. Cells were harvested for RNA isolation at 8, 12 and 16 h post infection (hpi). Total RNA was extracted from the cells using RNAiso plus (Takara, 9108) according to the manufacturer's instructions. The RNA was immediately reverse-transcribed into cDNA with the ExonScipt RT SuperMix with dsDNase (Exongen, A502-02) according to the manufacturer's instructions. The reaction contained in a final volume of 20 μl: Fast SYBR Green PCR Master Mix UDG (10 μl), each primer set (1 μl), and purified cDNA (1 μl), RNase-free water (7 μl). The RT-PCR cycling was 2 min at 50 °C (UDG pre-treatment), 10 min at 94 °C (enzyme activation), and then 3 min at 95 °C (initial denaturation), followed by 40 cycles of 95 °C for 5 s (denaturation), 54 °C for 30 s (annealing/extension). A melting curve of the real-time quantitative products (55–95 °C) was also obtained to ensure the absence of artifacts. Expression of β-actin was used to normalize the differences in total cDNA levels in the samples. The primers specific to IFN and ISGs used in PCR assays are shown in Table S10.

*Western Blot Analysis:* The PK-15 cells were seeded into 150 mm Cell Culture Dishes. When grown to 80–90 % confluence, the cells were infected with the PRV with MOI of 1 for 1 h and then washed with PBS twice and treated or not treated with NA-Fe for 8 h. The total protein was extracted using a commercial kit (Solarbio, BC3710) at 8 hpi. Lysates were mixed with SDS-PAGE Sample Loading Buffer (Biosharp, BL502B) and heated at 95 °C for 5min, followed by separation using SDS-PAGE under reducing conditions and blotting onto 0.2 μm PVDF membranes (Bio-Rad). After blocked with 5 % BSA for 1 h, proteins were stained using primary antibodies directed against OAS1 (Proteintech,14955-1-AP), MX1(Proteintech,13750-1-AP), ISG15 (Proteintech, 15981-1-AP), IRF3 (Proteintech,11312-1-AP), p-IRF3 (Ser386) (Affinity, AF3438), p-PKR (Abcam, ab81303). The secondary antibodies used for the western blot were Goat anti-rabbit IgG (Proteintech, SA00001-2). The results were developed by an ECL detection kit (Biosharp, BL520A) and band intensities were quantified using Image J.

*Application of NA-Fe in Pig farm fences and air purifier:* Based on materials of traditional pig farm fences, such as iron, aluminum, steel and plastic, 0, 0.0625, 0.125 and 0.25 mg cm^−2^ NA-Fe were loaded on the surface of the selected area (0.2 cm^2^). For the HEPA air purifier, 0, 0.0625, 0.125 and 0.25 mg cm^−2^ NA-Fe were loaded on the surface of fiberglass membranes (0.2 cm^2^). After drying under airflow at 37 °C, virus was sprayed onto the outermost layer. After incubation for 1 h, both the control area (equal volume PBS) and the NA-Fe area were placed into a tube containing 200 μL PBS. After washing, extrusion and centrifugation, viral suspensions were harvested, and followed by plaque assay.

*Statistical analysis:* All the statistical analyses were performed using GraphPad Prism 8 software (GraphPad Software). All data represent mean value ± standard deviation. The statistical significance of the data was assessed using a two-tailed Student's t-test with GraphPad Prism 8.0. Correlation analyses were evaluated by Pearson r^2^, ns: p > 0.05, ∗p < 0.05, ∗∗p < 0.01, and ∗∗∗p < 0.001.

## CRediT authorship contribution statement

**Hongping Wan:** Writing – review & editing, Writing – original draft, Supervision, Project administration, Methodology, Investigation, Funding acquisition, Conceptualization. **Zhengqun Huang:** Writing – original draft, Validation, Supervision, Software, Methodology, Investigation, Formal analysis, Data curation. **Mingrun Tang:** Writing – original draft, Methodology, Investigation, Funding acquisition, Formal analysis, Data curation. **Huirong Tan:** Methodology, Investigation, Formal analysis, Data curation. **Kai Deng:** Methodology, Investigation. **Yingnan Liu:** Methodology. **Xinghong Zhao:** Writing – review & editing, Writing – original draft, Supervision, Project administration, Methodology, Investigation, Conceptualization. **Hongjun Chen:** Conceptualization, Project administration, Supervision, Writing – review & editing.

## Declaration of competing interest

The authors declare that they have no known competing financial interests or personal relationships that could have appeared to influence the work reported in this paper.

## Data Availability

Data will be made available on request.

## References

[1] Cagno V., Andreozzi P., D'Alicarnasso M., Silva P.J., Mueller M., Galloux M., Le Goffic R., Jones S.T., Vallino M., Hodek J., Weber J., Sen S., Janecek E.R., Bekdemir A., Sanavio B., Martinelli C., Donalisio M., Welti M.A.R., Eleouet J.F., Han Y., Kaiser L., Vukovic L., Tapparel C., Král P., Krol S., Lembo D., Stellacci F. (2018). Broad-spectrum non-toxic antiviral nanoparticles with a virucidal inhibition mechanism. Nat. Mater..

[2] Prakash J., Cho J., Mishra Y.K. (2022). Photocatalytic TiO2 nanomaterials as potential antimicrobial and antiviral agents: scope against blocking the SARS-COV-2 spread. Micro Nano Eng..

[3] Checconi P., Mariconda A., Catalano A., Ceramella J., Pellegrino M., Aquaro S., Sinicropi M.S., Longo P. (2025). Searching for new gold (I)-Based complexes as anticancer and/or antiviral agents. Molecules.

[4] Luceri A., Francese R., Lembo D., Ferraris M., Balagna C. (2023). Silver nanoparticles: review of antiviral properties, mechanism of action and applications. Microorganisms.

[5] Lin C.J., Chang L., Chu H.W., Lin H.J., Chang P.C., Wang R.Y.L., Unnikrishnan B., Mao J.Y., Chen S.Y., Huang C.C. (2019). High amplification of the antiviral activity of curcumin through transformation into carbon quantum dots. Small.

[6] Xu X., Zhang J., Liu S., Wang C., Wang H., Fan H., Tong Y., Liu H., Zhou D. (2022). New advances in nanomaterial‐based antiviral strategies. Small Struct..

[7] Xia Y., Xiao M., Zhao M., Xu T., Guo M., Wang C., Li Y., Zhu B., Liu H. (2020). Doxorubicin-loaded functionalized selenium nanoparticles for enhanced antitumor efficacy in cervical carcinoma therapy. Mater. Sci. Eng. C.

[8] Tavakoli A., Hashemzadeh M.S. (2020). Inhibition of herpes simplex virus type 1 by copper oxide nanoparticles. J. Virol. Methods.

[9] Li B., Ma R., Chen L., Zhou C., Zhang Y.X., Wang X., Huang H., Hu Q., Zheng X., Yang J., Shao M., Hao P., Wu Y., Che Y., Li C., Qin T., Gao L., Niu Z., Li Y. (2023). Diatomic iron nanozyme with lipoxidase-like activity for efficient inactivation of enveloped virus. Nat. Commun..

[10] Qin T., Chen Y., Miao X., Shao M., Xu N., Mou C., Chen Z., Yin Y., Chen S., Yin Y., Gao L., Peng D., Liu X. (2024). Low-Temperature adaptive single-atom iron nanozymes against viruses in the cold chain. Adv. Mater..

[11] Miao X., Yin Y., Chen Y., Bi W., Yin Y., Chen S., Peng D., Gao L., Qin T., Liu X. (2023). Bidirectionally regulating viral and cellular ferroptosis with metastable iron sulfide against influenza virus. Adv. Sci..

[12] Wu J., Wang X., Wang Q., Lou Z., Li S., Zhu Y., Qin L., Wei H. (2019). Nanomaterials with enzyme-like characteristics (nanozymes): Next-generation artificial enzymes (II). Chem. Soc. Rev..

[13] Qin T., Ma R., Yin Y., Miao X., Chen S., Fan K., Xi J., Liu Q., Gu Y., Yin Y., Hu J., Liu X., Peng D., Gao L. (2019). Catalytic inactivation of influenza virus by iron oxide nanozyme. Theranostics.

[14] Li R., Wang L. (2019). Baicalin inhibits influenza virus A replication via activation of type I IFN signaling by reducing miR-146a. Mol. Med. Rep..

[15] Shi H., Ren K., Lv B., Zhang W., Zhao Y., Tan R.X., Li E. (2016). Baicalin from Scutellaria baicalensis blocks respiratory syncytial virus (RSV) infection and reduces inflammatory cell infiltration and lung injury in mice. Sci. Rep..

[16] Moghaddam E., Teoh B.T., Sam S.S., Lani R., Hassandarvish P., Chik Z., Yueh A., Abubakar S., Zandi K. (2014). Baicalin, a metabolite of baicalein with antiviral activity against dengue virus. Sci. Rep..

[17] Ren C.Z., Hu W.Y., Zhang J.W., Wei Y.Y., Yu M.L., Hu T.J. (2021). Establishment of inflammatory model induced by Pseudorabies virus infection in mice. J. Vet. Sci..

[18] Villalobos-Sánchez E., García-Ruiz D., Camacho-Villegas T.A., Canales-Aguirre A.A., Gutiérrez-Ortega A., Muñoz-Medina J.E., Elizondo-Quiroga D.E. (2023). In vitro antiviral activity of nordihydroguaiaretic acid against SARS-CoV-2. Viruses.

[19] Merino-Ramos T., Jiménez de Oya N., Saiz J.-C., Martín-Acebes M.A. (2017). Antiviral activity of nordihydroguaiaretic acid and its derivative tetra-O-methyl nordihydroguaiaretic acid against West Nile virus and Zika virus. Antimicrob. Agents Chemother..

[20] Wang Y., Zhou J., Yuan L., Wu F., Xie L., Yan X., Li H., Li Y., Shi L., Hu R., Liu Y. (2023). Neighboring carboxylic acid boosts peroxidase-like property of metal-phenolic nano-networks in eradicating Streptococcus mutans biofilms. Small.

[21] Chen Y., Yang X., Li H., Wu X., Wu W., Chen J., Wu A., Wang X. (2024). Self-Assembled Fe-Phenolic acid network synergizes with ferroptosis to enhance tumor nanotherapy. Small.

[22] Liaw J.-W., Tsai S.-W., Lin H.-H., Yen T.-C., Chen B.-R. (2012). Wavelength-dependent Faraday–Tyndall effect on laser-induced microbubble in gold colloid. J. Quant. Spectrosc. Radiat. Transf..

[23] Yuan K., Sun Y., Liang F., Pan F., Hu M., Hua F., Yuan Y., Nie J., Zhang Y. (2022). Tyndall-effect-based colorimetric assay with colloidal silver nanoparticles for quantitative point-of-care detection of creatinine using a laser pointer pen and a smartphone. RSC Adv..

[24] Wei H., Wang E. (2013). Nanomaterials with enzyme-like characteristics (nanozymes): Next-generation artificial enzymes. Chem. Soc. Rev..

[25] Cao S., Tao F.F., Tang Y., Li Y., Yu J. (2016). Size- and shape-dependent catalytic performances of oxidation and reduction reactions on nanocatalysts. Chem. Soc. Rev..

[26] Kuang Y., Wang Q., Chen Z., Megharaj M., Naidu R. (2013). Heterogeneous Fenton-like oxidation of monochlorobenzene using green synthesis of iron nanoparticles. J. Colloid Interface Sci..

[27] V Sithara N., Bharathi D., Lee J., Mythili R., Devanesan S., AlSalhi M.S. (2024). Synthesis of iron oxide nanoparticles using orange fruit peel extract for efficient remediation of dye pollutant in wastewater. Environ. Geochem. Health.

[28] Haeggström J.Z., Funk C.D. (2011). Lipoxygenase and leukotriene pathways: biochemistry, biology, and roles in disease. Chem. Rev..

[29] Sirijaraensre J., Limtrakul J. (2013). Structures and mechanisms of the dehydration of benzaldoxime over Fe-ZSM-5 zeolites: a DFT study. Struct. Chem..

[30] Correa Sierra C.B., Schang L.M. (2022). On the sensitivity of the virion envelope to lipid peroxidation. Microbiol. Spectr..

[31] Imani S.M., Ladouceur L., Marshall T., Maclachlan R., Soleymani L., Didar T.F. (2020). Antimicrobial nanomaterials and coatings: current mechanisms and future perspectives to control the spread of viruses including SARS-CoV-2. ACS Nano.

[32] Chen J., huan Hu J., cong Sun R., han Li X., Zhou J., Zhou B. (2023). Porcine Mx proteins inhibit pseudorabies virus replication through interfering with early gene synthesis. Vet. Microbiol..

[33] Lee E.C., Davis-Poynter N., Nguyen C.T.H., Peters A.A., Monteith G.R., Strounina E., Popat A., Ross B.P. (2016). GAG mimetic functionalised solid and mesoporous silica nanoparticles as viral entry inhibitors of herpes simplex type 1 and type 2 viruses. Nanoscale.

[34] Su H., Yao S., Zhao W., Li M., Liu J., Shang W.J., Xie H., Ke C., Gao M., Yu K., Liu H., Shen J., Tang W., Zhang L., Zuo J., Jiang H., Bai F., Wu Y., Ye Y., Xu Y. (2020). Discovery of baicalin and baicalein as novel, natural product inhibitors of SARS-CoV-2 3CL protease in vitro. bioRxiv.

[35] Zhu J., Zhang Y., Ghosh A., Cuevas R.A., Forero A., Dhar J., Ibsen M.S., Schmid-Burgk J.L., Schmidt T., Ganapathiraju M.K., Fujita T., Hartmann R., Barik S., Hornung V., Coyne C.B., Sarkar S.N. (2014). Antiviral activity of human OASL protein is mediated by enhancing signaling of the RIG-I RNA sensor. Immunity.

[36] Imaizumi T., Yoshida H., Hayakari R., Xing F., Wang L., Matsumiya T., Tanji K., Kawaguchi S., Murakami M., Tanaka H. (2016). Interferon-stimulated gene (ISG) 60, as well as ISG56 and ISG54, positively regulates TLR3/IFN-β/STAT1 axis in U373MG human astrocytoma cells. Neurosci. Res..

[37] Lenschow D.J., Lai C., Frias-Staheli N., Giannakopoulos N.V., Lutz A., Wolff T., Osiak A., Levine B., Schmidt R.E., García-Sastre A., Leib D.A., Pekosz A., Knobeloch K.P., Horak I., Virgin H.W. (2007). IFN-stimulated gene 15 functions as a critical antiviral molecule against influenza, herpes, and Sindbis viruses. Proc. Natl. Acad. Sci. USA..

[38] Melchjorsen J., Kristiansen H., Christiansen R., Rintahaka J., Matikainen S., Paludan S.R., Hartmann R. (2009). Differential regulation of the OASL and OAS1 genes in response to viral infections. J. Interferon Cytokine Res..

[39] Yang X., Liu X., Nie Y., Zhan F., Zhu B. (2023). Oxidative stress and ROS-mediated cellular events in RSV infection: potential protective roles of antioxidants. Virol. J..

[40] Ting D., Dong N., Fang L., Lu J., Bi J., Xiao S., Han H. (2018). Multisite inhibitors for enteric coronavirus: antiviral cationic carbon dots based on curcumin. ACS Appl. Nano Mater..

[41] Pan Y., Qin R., Hou M., Xue J., Zhou M., Xu L., Zhang Y. (2022). The interactions of polyphenols with Fe and their application in Fenton/Fenton-like reactions. Sep. Purif. Technol..

[42] Ye S., Shao K., Li Z., Guo N., Zuo Y., Li Q., Lu Z., Chen L., He Q., Han H. (2015). Antiviral activity of graphene oxide: how sharp edged structure and charge matter. ACS Appl. Mater. Interfaces.

[43] Kumar R., Nayak M., Sahoo G.C., Pandey K., Sarkar M.C., Ansari Y., Das V.N.R., Topno R.K., Bhawna, Madhukar M., Das P. (2019). Iron oxide nanoparticles based antiviral activity of H1N1 influenza A virus. J. Infect. Chemother..

[44] Innocenzi P., Stagi L. (2020). Carbon-based antiviral nanomaterials: Graphene, C-dots, and fullerenes. A perspective. Chem. Sci..

[45] Neese F. (2018). Software update: the ORCA program system, version 4.0. Wiley Interdiscip. Rev. Comput. Mol. Sci..

[46] Neese F. (2012). The ORCA program system. Wiley Interdiscip. Rev. Comput. Mol. Sci..

[47] Neese F. (2022). Software update: the ORCA program system—Version 5.0. Wiley Interdiscip. Rev. Comput. Mol. Sci..

[48] Lu T., Chen F. (2012). Atomic dipole moment corrected Hirshfeld population method. J. Theor. Comput. Chem..

[49] Klein J., Khartabil H., Boisson J.-C., Contreras-García J., Piquemal J.-P., Hénon E. (2020). New way for probing bond strength. J. Phys. Chem..

[50] Niu Q., Huang Q., Yu T.-Y., Liu J., Shi J.-W., Dong L.-Z., Li S.-L., Lan Y.-Q. (2022). Achieving high photo/thermocatalytic product selectivity and conversion via thorium clusters with switchable functional ligands. J. Am. Chem. Soc..

[51] Lu T. (2024). A comprehensive electron wavefunction analysis toolbox for chemists, Multiwfn. J. Chem. Phys..

[52] Lu T., Chen F. (2012). Multiwfn: a multifunctional wavefunction analyzer. J. Comput. Chem..

[53] Humphrey W., Dalke A., Schulten K. (1996). VMD: visual molecular dynamics. J. Mol. Graph..

[54] Weigend F., Ahlrichs R. (2005). Balanced basis sets of split valence, triple zeta valence and quadruple zeta valence quality for H to Rn: design and assessment of accuracy. Phys. Chem. Chem. Phys..

[55] Grimme S., Ehrlich S., Goerigk L. (2011). Effect of the damping function in dispersion corrected density functional theory. J. Comput. Chem..

[56] Grimme S., Antony J., Ehrlich S., Krieg H. (2010). A consistent and accurate ab initio parametrization of density functional dispersion correction (DFT-D) for the 94 elements H-Pu. J. Chem. Phys..

[57] Mardirossian N., Head-Gordon M. (2014). ωB97X-V: a 10-parameter, range-separated hybrid, generalized gradient approximation density functional with nonlocal correlation, designed by a survival-of-the-fittest strategy. Phys. Chem. Chem. Phys..

[58] Weigend F. (2006). Accurate Coulomb-fitting basis sets for H to Rn. Phys. Chem. Chem. Phys..

[59] Neese F., Wennmohs F., Hansen A., Becker U. (2009). Efficient, approximate and parallel Hartree–Fock and hybrid DFT calculations. A ‘chain-of-spheres’ algorithm for the Hartree–Fock exchange. Chem. Phys..

