# Supplementary material for: Harnessing nanozyme-immunomodulation for antiviral defense via Fe–nordihydroguaiaretic acid nano-networks
Source: Mater Today Bio. 2025 Nov 20;35:102567. doi: 10.1016/j.mtbio.2025.102567 (PMC12719199; doi:10.1016/j.mtbio.2025.102567)
Supplement: Multimedia component 1 [file mmc1.docx]

**Harnessing Nanozyme-Driven Immunomodulation for Antiviral Defense via Fe–Nordihydroguaiaretic Acid Nano-Networks**

Hongping Wan^a,1,^ *, Zhengqun Huang ^a,1^, Mingrun Tang ^a^, Huirong Tan ^a^, Kai Deng ^a^, Yingnan Liu ^b^, Xinghong Zhao ^a,b^ *, Hongjun Chen ^b,^ *

^a^ Center for Infectious Diseases Control (CIDC), Sichuan Agricultural University, Chengdu, 611130, China.

^b^ State Key Laboratory of Veterinary Public Health and Safety, College of Veterinary Medicine, China Agricultural University, Beijing 100193, China

^1^Author contribute equally and share the first author

* Correspondence: [x.zhao@cau.edu.cn](mailto:x.zhao@cau.edu.cn) (X. Zhao); [hpwan508@126.com](mailto:hpwan508@126.com) (H. Wan), [vetchj@cau.edu.cn](mailto:vetchj@cau.edu.cn) (H. Chen)


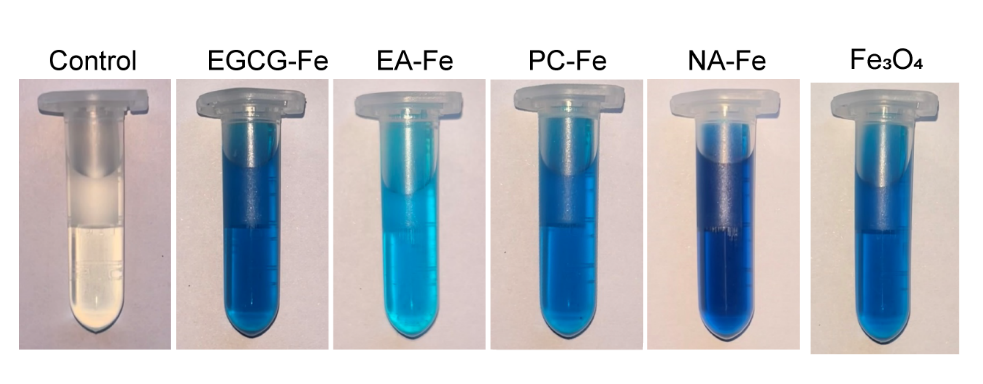


Figure S1. Bright field image of metal-phenolic nano-networks (0.1 mg/mL) catalyzed H_2_O_2_ reaction with TMB (20 mM) for 10 min.


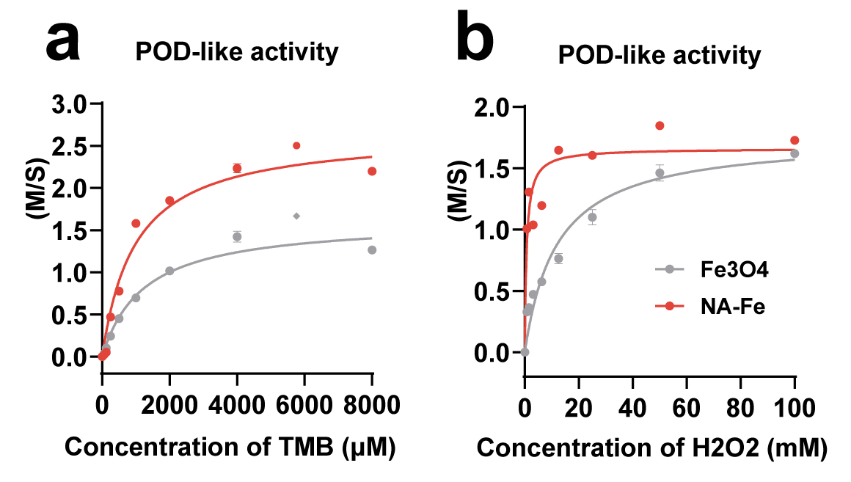


Figure S2. Michaelis-Menten kinetics for peroxidase-like activity of NA-Fe and Fe_3_O_4_ towards a) TMB and b) H_2_O_2_.

Table S1. Kinetic parameters of NA-Fe and Fe_3_O_4_ when H_2_O_2_ concentration was variable

| **H_2_O_2_ substrate** | **Km ( mM)** | **Vmax (nM/S)** |
| --- | --- | --- |
| Fe_3_O_4_ | 11.96 | 175.6 |
| NA-Fe | 0.7029 | 166.1 |

Table S2. Kinetic parameters of NA-Fe and Fe_3_O_4_ when TMB concentration was variable

| **TMB substrate** | **Km (mM)** | **Vmax (nM/S)** |
| --- | --- | --- |
| Fe_3_O_4_ | 1.237 | 162.1 |
| NA-Fe | 0.9805 | 265.9 |


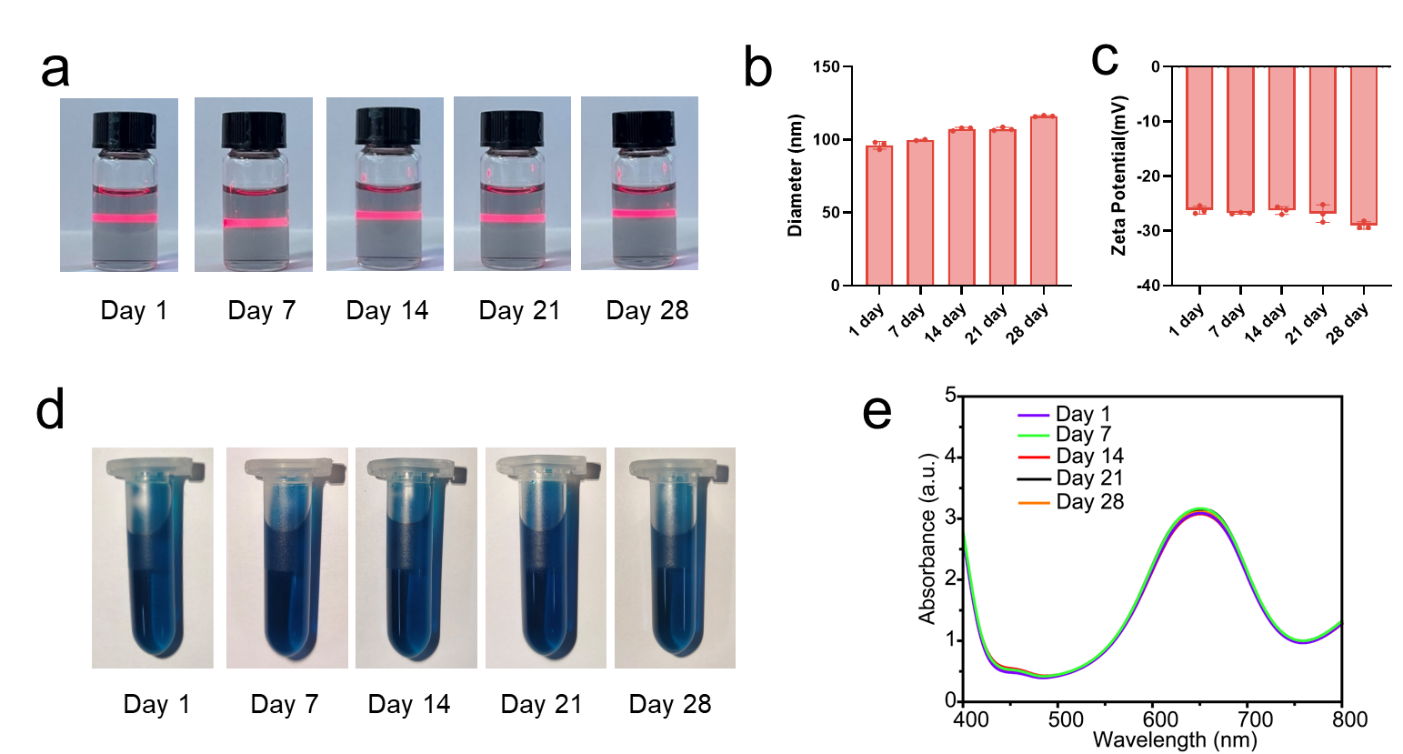


Figure S3. 28-day stability test of NA-Fe. a) The pictures of Tyndall effect of NA-Fe taken weekly for 28 days. b) Diameter distribution and c) Zeta potentials of NA-Fe measured weekly for 28 days. d) Bright field image of NA-Fe (0.1 mg/mL) catalyzed H_2_O_2_ reaction with TMB (20 mM) for 10 min. e) UV−vis absorption of TMB after the treatments with different NA-Fe for 10 min in the presence of 1 mM H_2_O_2_.


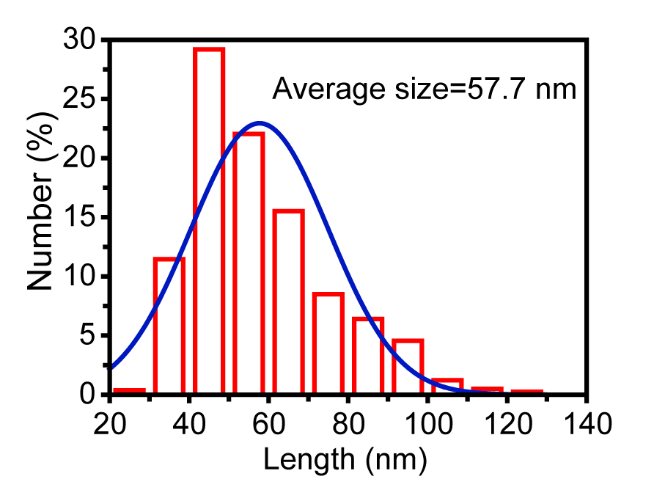


Figure S4. Length distribution and aspect ratio analysis of NA-Fe.

Table S3. EXAFS data fitting results of Samples.

| Sample | Shell | *N^a^* | *R*(Å)*^b^* | *σ*^2^(Å^2^)*^c^* | Δ*E*_0_ (eV)*^d^* | *R* factor |
| --- | --- | --- | --- | --- | --- | --- |
| Fe foil | Fe-Fe | 8* | 2.47±0.01 | 0.0053 | 6.1±1.2 | 0.0043 |
|  | Fe-Fe | 6* | 2.85±0.01 | 0.0066 |  |  |
| NA-Fe | Fe-O | 6.2±0.3 | 2.00±0.01 | 0.0074 | -1.4±1.2 | 0.0097 |
|  | Fe-Fe | 1.8±0.8 | 2.84±0.03 | 0.0154 |  |  |
|  | Fe-Fe | 1.8±0.9 | 3.16±0.03 | 0.0130 |  |  |

*^a^N*: coordination numbers; *^b^R*: bond distance; *^c^σ*^2^: Debye-Waller factors; *^d^* Δ*E*_0_: the inner potential correction. *R* factor: goodness of fit. *Ѕ*_0_^2^ was set to 0.81, according to the experimental EXAFS fit of Fe foil reference by fixing CN as the known crystallographic value.

Table S4. HRMS molecular weight of NA-Fe.

| **Molecular formula** | **Measured value (m/z)** | **Calculated value (m/z)** | **Relative error (m/z)** |
| --- | --- | --- | --- |
| [FeC_18_O_4_ H_20_·4H_2_O]^+^ | 437.2 | 428.2 | -9 |

Table S5. Fe ^3+^ release level from NA-Fe (5 mg/mL) in DMEM and H_2_O following a 10-day incubation

| Samples | *Fe* (μg/L) | *Fe* Release rate |
| --- | --- | --- |
| NA-Fe in DMEM | 1819.29±15.25 | <0.5% |
| NA-Fe in H_2_O | 346.08±2.18 | <0.09% |
| NA-Fe | 4.1×10^5^±1.22×10^3^ |  |


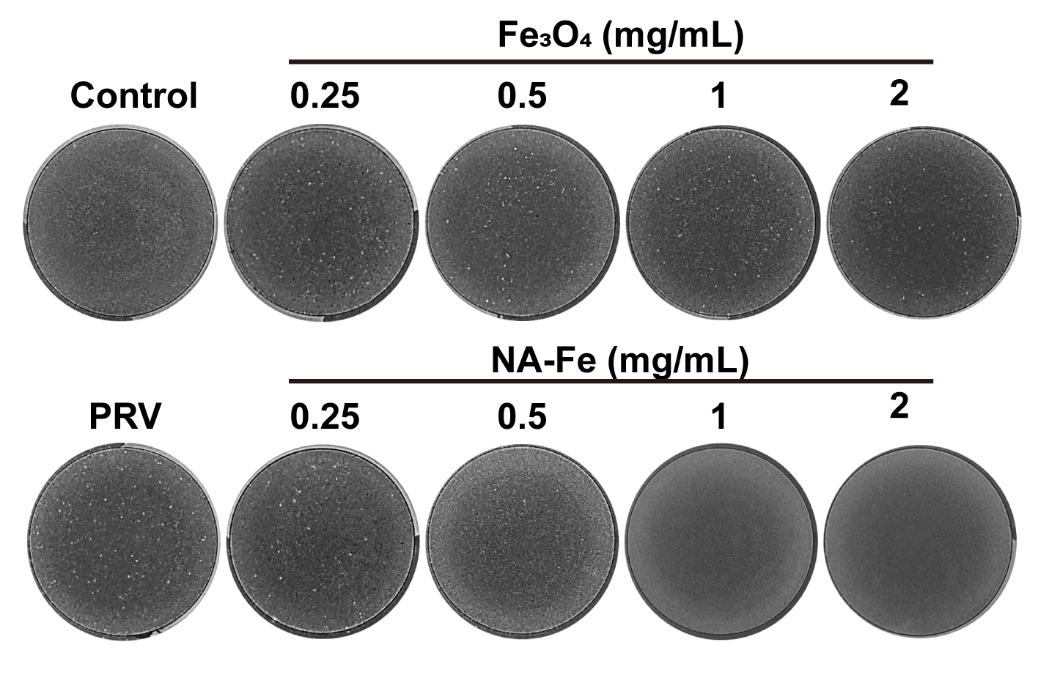


Figure S5. Bright-field image of viral plaque of PRV after with or without different concentration of NA-Fe and Fe_3_O_4_ treatment.


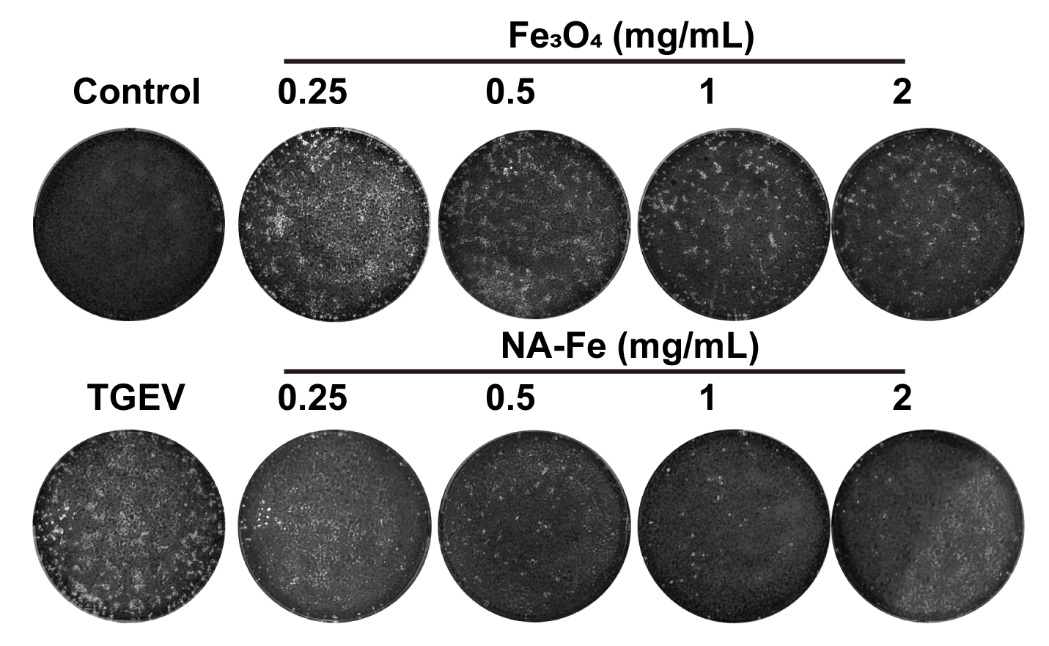


Figure S6. Bright-field image of viral plaque of TGEV after with or without different concentration of NA-Fe and Fe_3_O_4_ treatment.


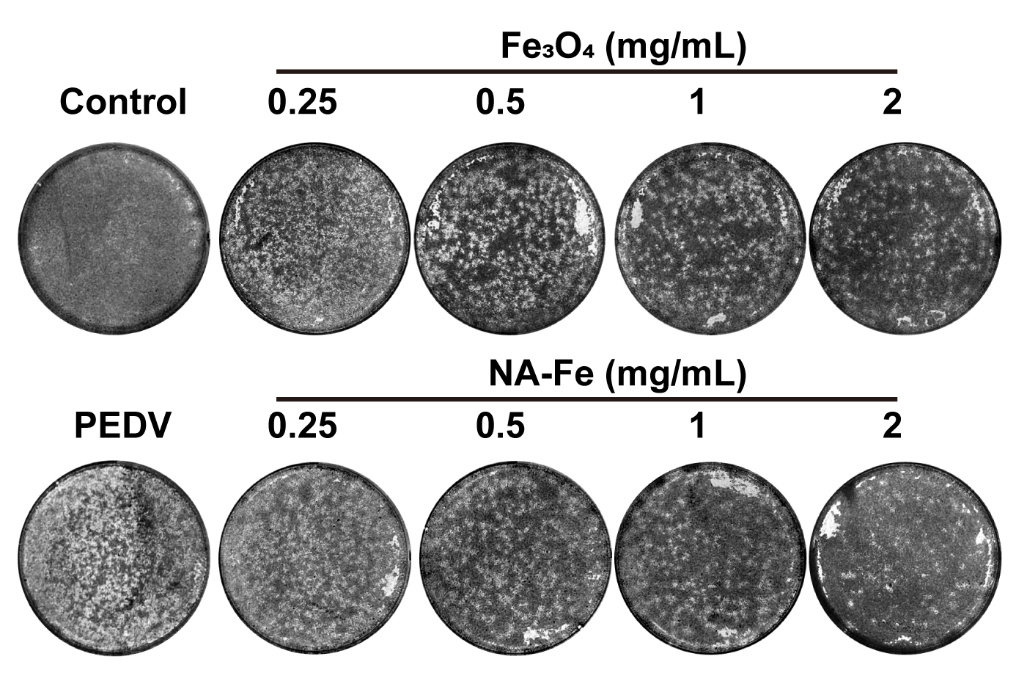


Figure S7. Bright-field image of viral plaque of PEDV after with or without different concentration of NA-Fe and Fe_3_O_4_ treatment.

**Table S6.** The ADCH charge of substrate.

| **Atom** | **ADCH Charge** |
| --- | --- |
| 44(C ) | -0.13483159 |
| 45(C ) | -0.09054101 |
| 46(C ) | -0.10175241 |
| 47(C ) | -0.11241975 |
| 48(C ) | -0.25010344 |
| 49(C ) | -0.25059815 |
| 50(C ) | -0.14419611 |
| 51(H ) | 0.13583991 |
| 52(H ) | 0.14241791 |
| 53(H ) | 0.16137133 |
| 54(H ) | 0.17339395 |
| 55(H ) | 0.18089270 |
| 56(H ) | 0.13463815 |
| 57(H ) | 0.10901257 |
| 58(H ) | 0.09092801 |
| 59(H ) | 0.12665068 |
| 60(H ) | 0.08967876 |
| 61(H ) | 0.10285374 |
| 62(H ) | 0.16768922 |

**Table S7.** The chemical bonds distance and intrinsic bond strength index of substrate before and after catalyst and substrate adsorption.

| Before catalyst and substrate adsorption | | |  | After catalyst and substrate adsorption | | |
| --- | --- | --- | --- | --- | --- | --- |
| Atom | Distance | IBSI |  | Atom | Distance | IBSI |
| 3(C ),4 (C ) | 1.3332 | 1.10617 |  | 46(C ),47 (C ) | 1.3712 | 1.05911 |
| 2(C ),7 (C ) | 1.3332 | 1.10750 |  | 45(C ),50 (C ) | 1.3813 | 1.02561 |


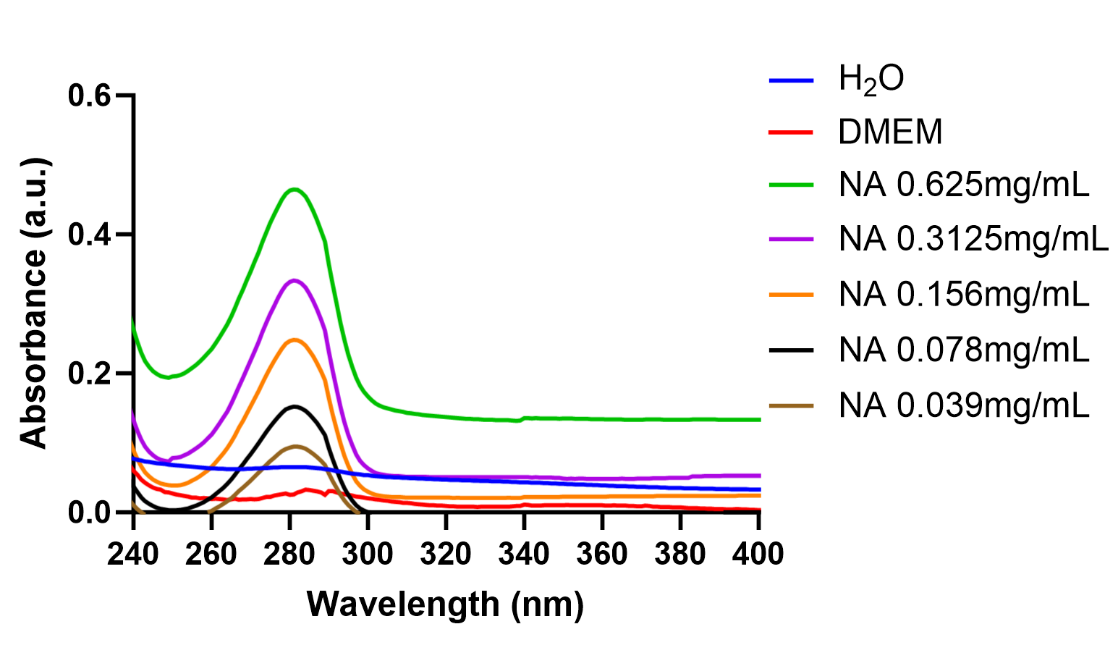


Figure S8. UV–vis spectra of NA released from NA–Fe in H_2_O and DMEM over a 10-day period. No distinct peak was observed at 280 nm, indicating that NA was not released from NA-Fe.


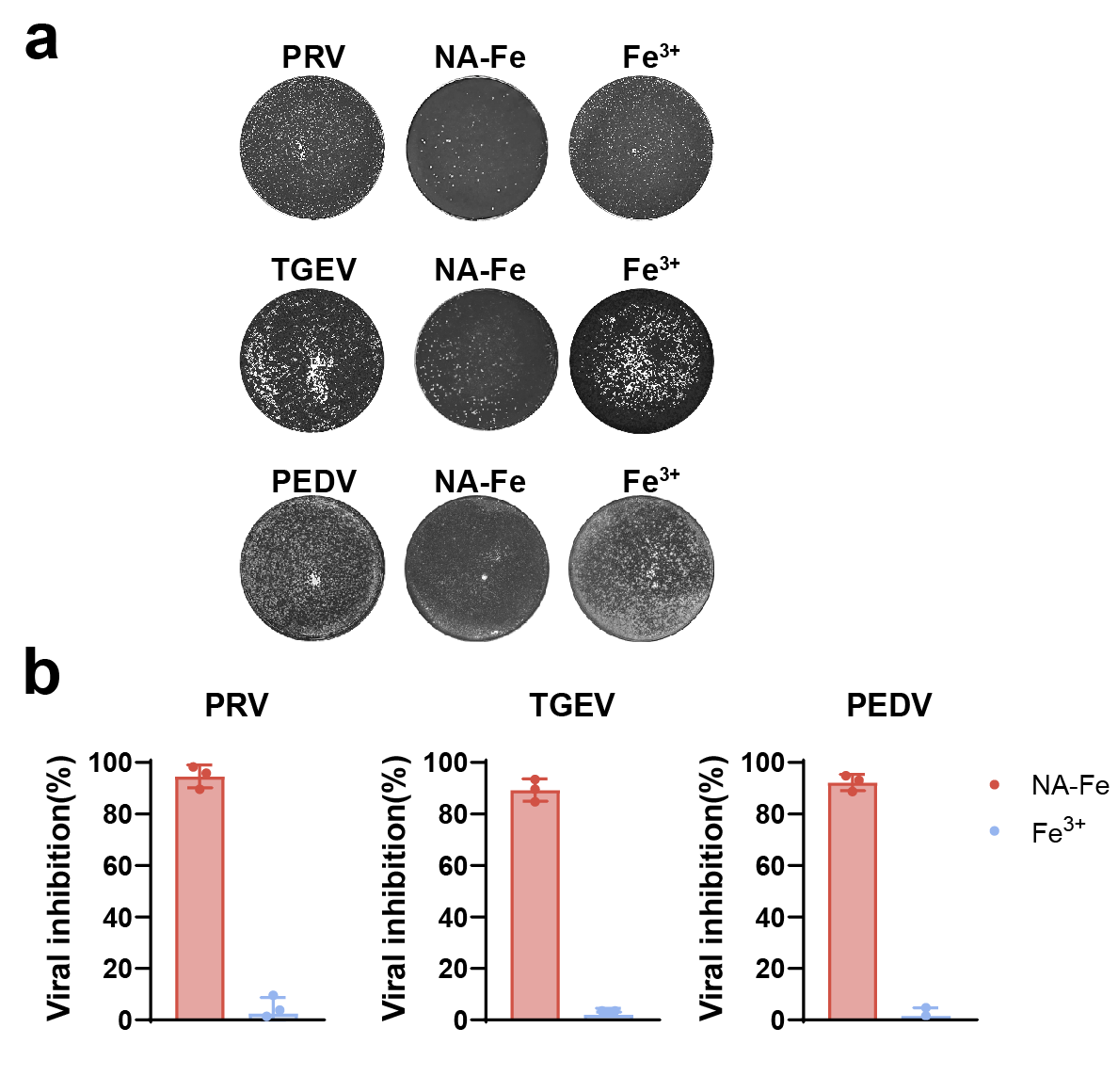


Figure S9. The antiviral activity of Fe^3+^ at the concentration of 2 μg/mL.


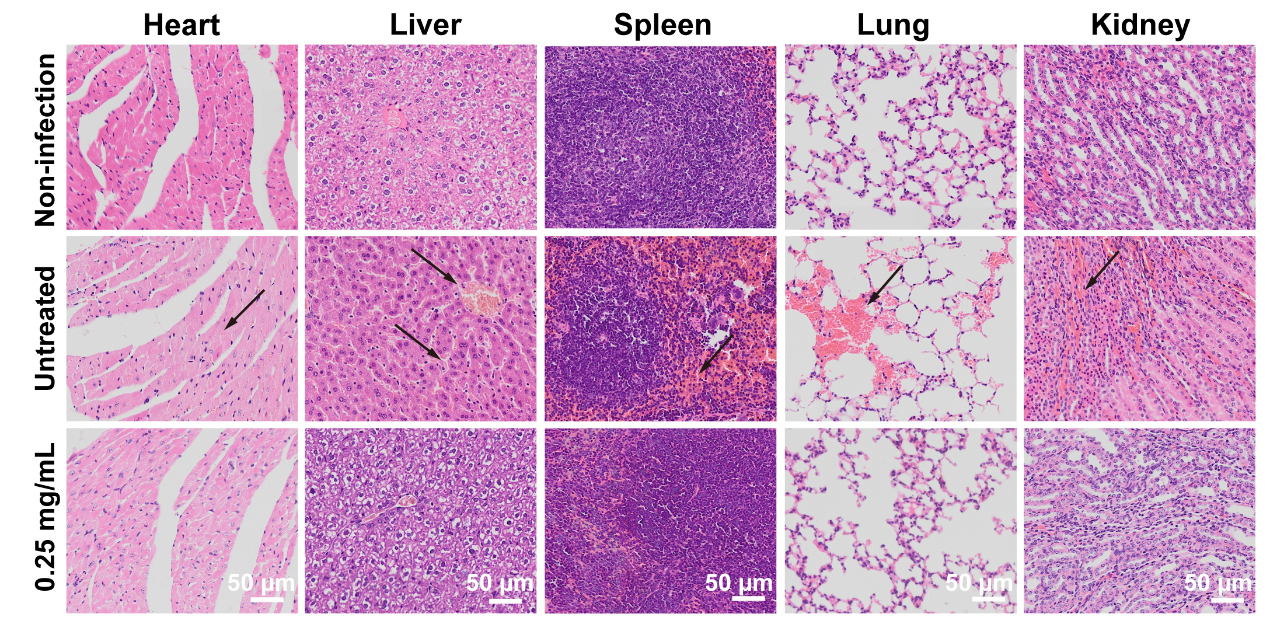


Figure S10. NA-Fe impaired the pathogenicity of PRV in vivo. Effect of NA-Fe (0.25 mg/mL) on the microstructures of lungs, spleens, heart, liver, and kidney of mice at 3 dpi (n=3).


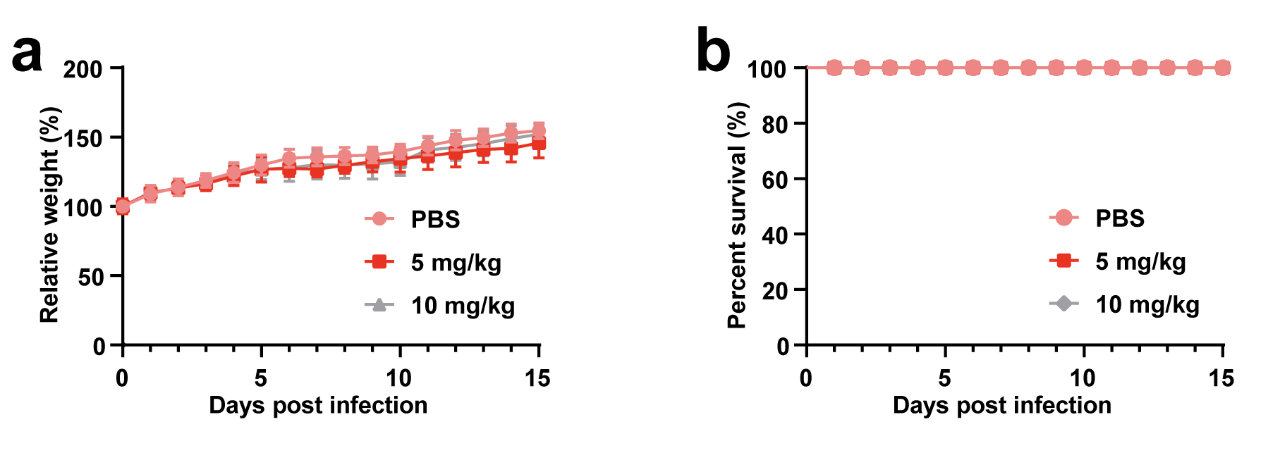


Figure S11. Biosafety assays of NA-Fe *in vivo*. a) Body weight and b) Survival rate of mice was monitored for 15 consecutive days (n=10).


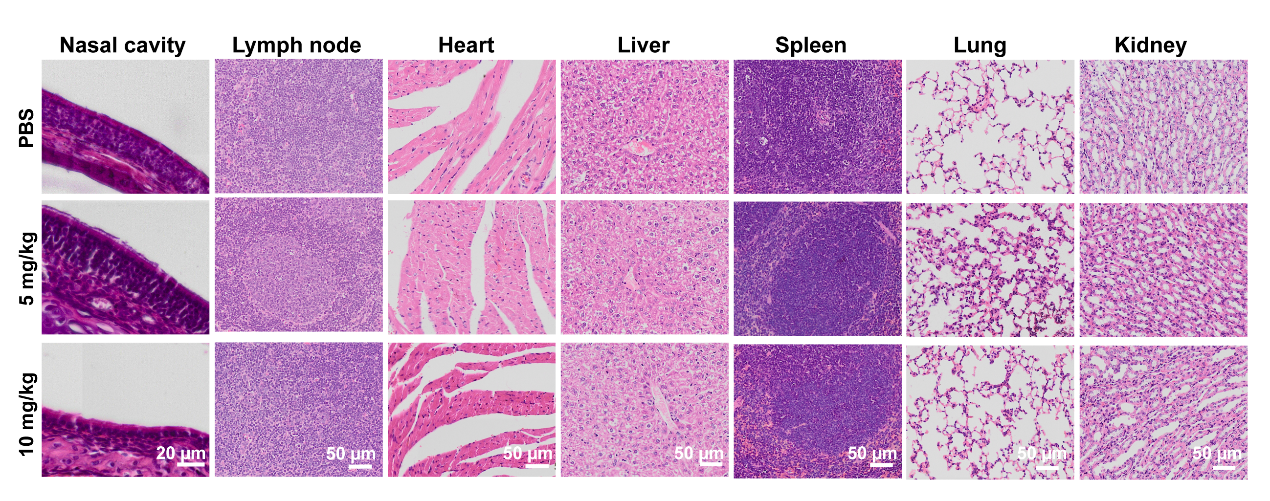


Figure S12. Biosafety assays of NA-Fe both in vivo. Effect of NA-Fe (5 mg/kg or 10 mg/kg) on the microstructures of nasal cavity, lymph node, lungs, spleens, heart, liver, and kidney of mice at 7 dpi (n=3).


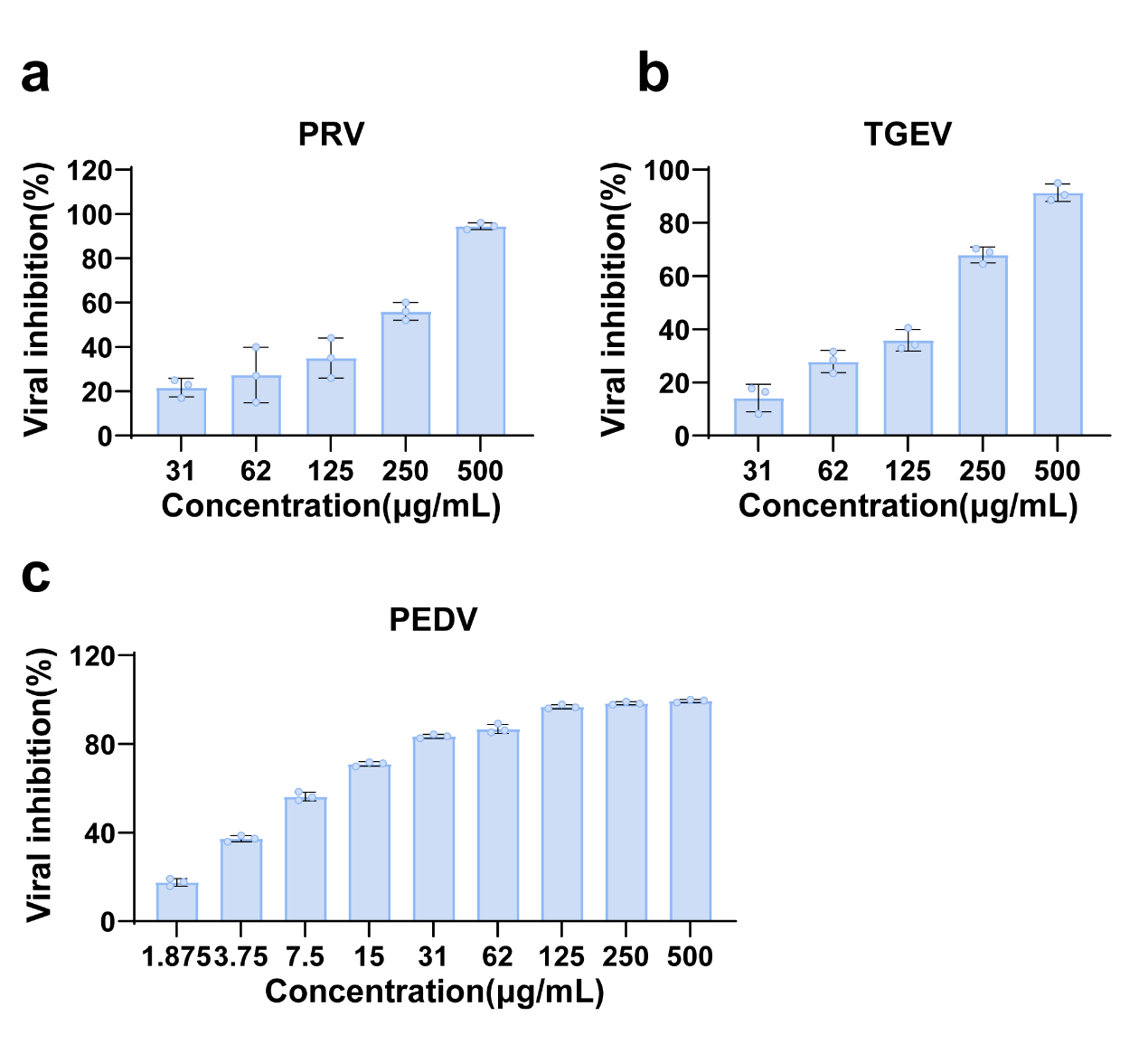


Figure S13. Inhibition of PRV, TGEV, and PEDV by different concentrations of NA-Fe.


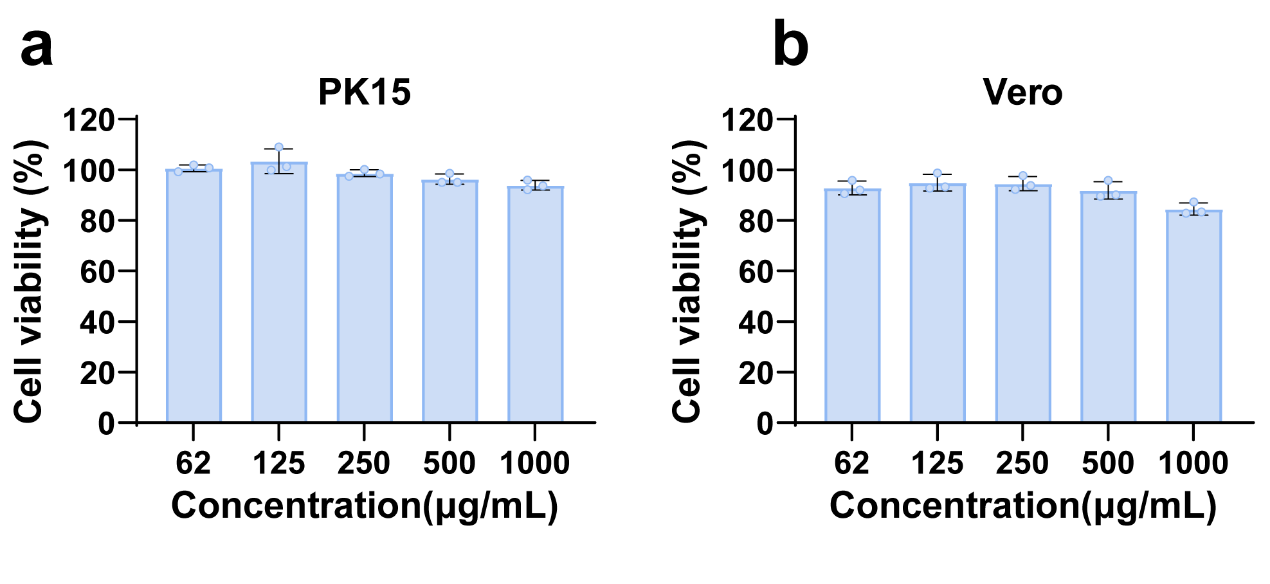


Figure S14. Biosafety assays of NA-Fe in vitro. Relative cell viability of PK-15 and Vero cells treated with NA-Fe at concentrations ranging from 62 to 1000 μg/mL.

Table S8. CC_50_, EC_50_, and SI of NA-Fe against PRV in PK-15 cells, TGEV in PK-15 cells, and PEDV in Vero cells.

| **Materials** | **PK-15** | **PRV** | |  | **TGEV** | |  | **Vero** | **PEDV** | |
| --- | --- | --- | --- | --- | --- | --- | --- | --- | --- | --- |
|  | **CC_50_**  **[μg mL^-1^]** | **EC_50_**  **[μg mL^-1^]** | **SI** |  | **EC_50_**  **[****μg mL^-1^]** | **SI** |  | **CC_50_**  **[μg mL^-1^]** | **EC_50_**  **[μg mL^-1^]** | **SI** |
| NA-Fe | >1000 | 172.4 | >5.8 |  | 169.6 | >5.9 |  | >1000 | 5.5 | >181.8 |


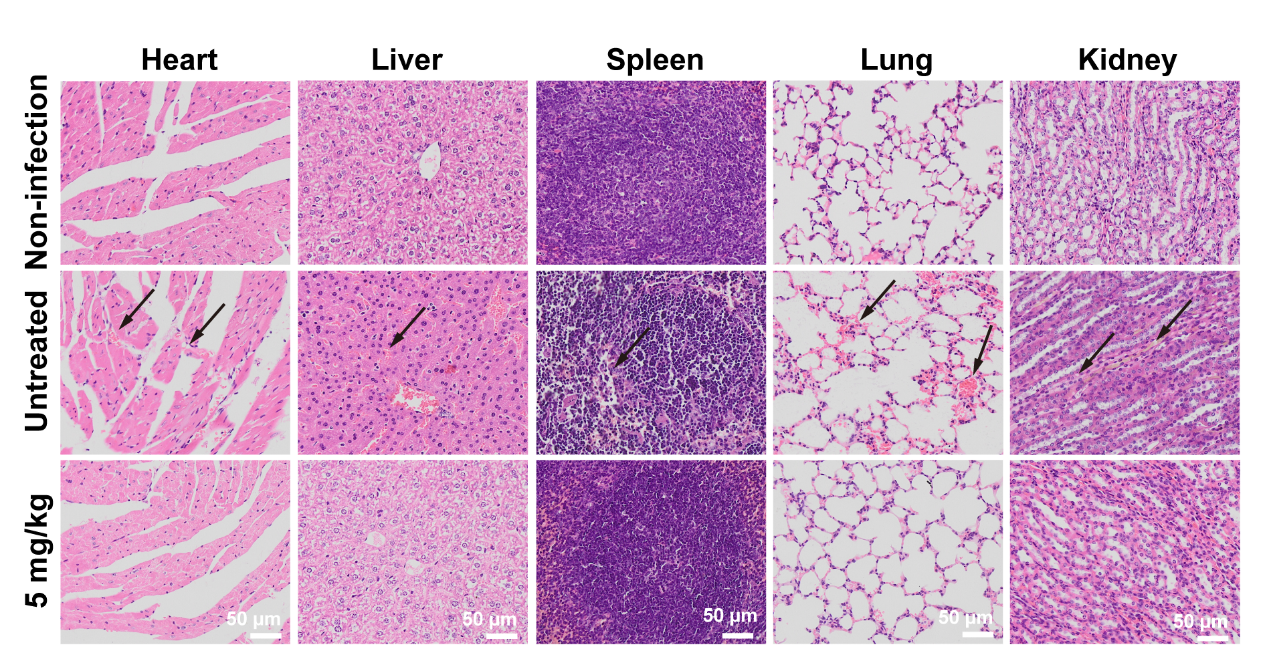


Figure S15. Effect of NA-Fe (5 mg/kg) on the microstructures of brains of mice at 3 dpi (n=3).


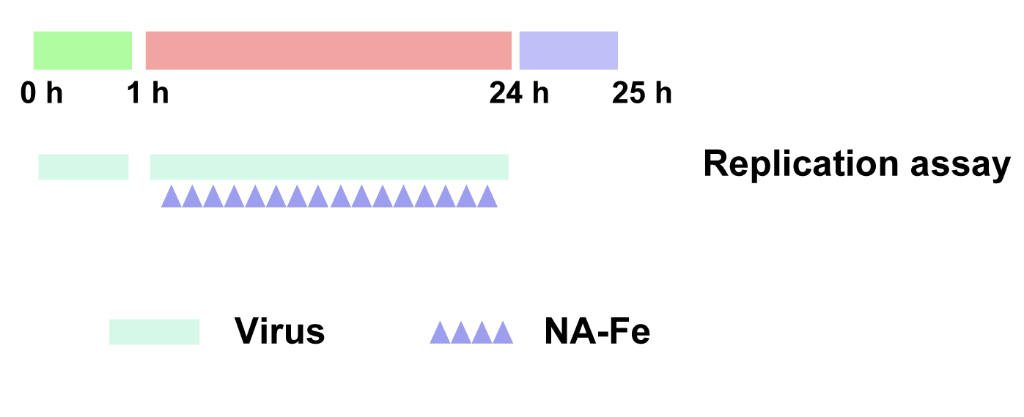


Figure S16. Flow chart of replication-stage inhibition of NA-Fe on virus proliferation.


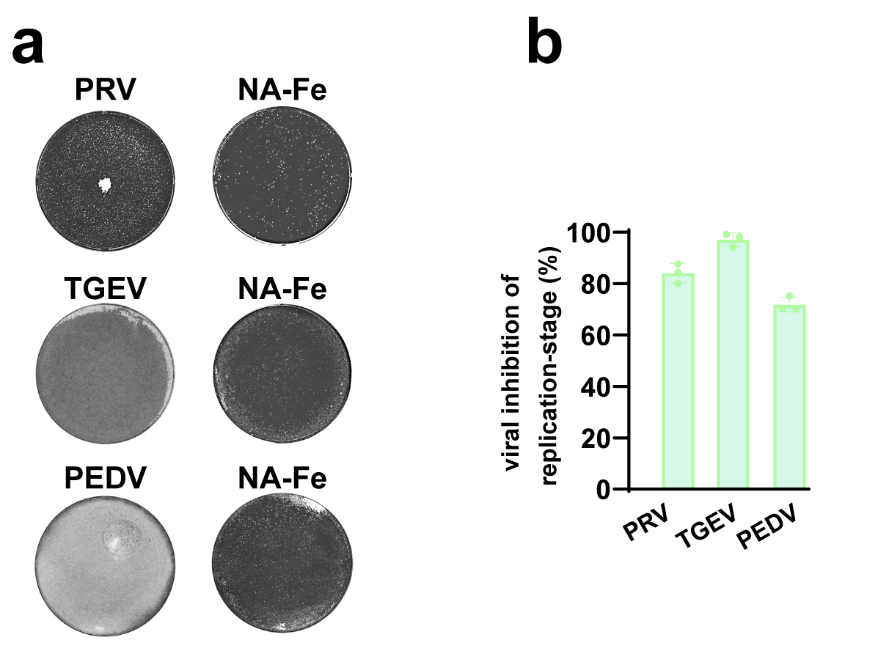


Figure S17. Bright-field image of viral plaque after with or without NA-Fe (0.5 mg/mL) treatment.


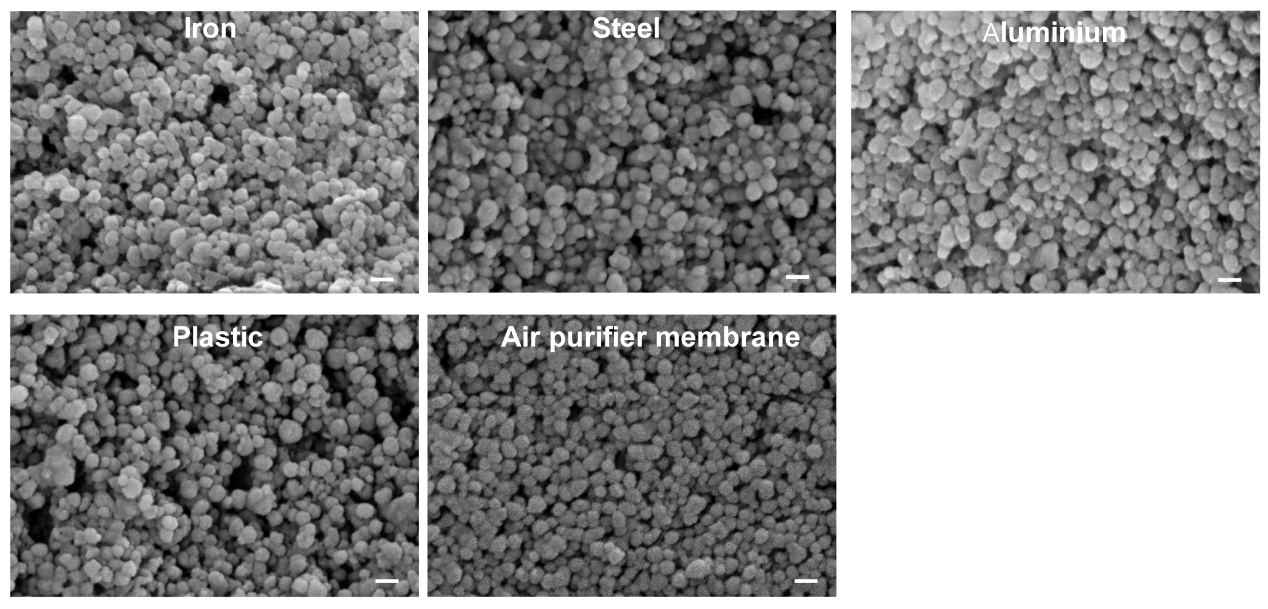


Figure S18. NA-Fe can be evenly applied to fences and air purifiers on pig farms. Bars represent 100 nm.

Table S9. Probe and primers used for FQ-PCR assay.

| Probe and Primers | | |
| --- | --- | --- |
| gB probe |  | 5′-ACGTCATCGTCACGACC-3′ |
| Primers | gB-F | ACAAGTTCAAGGCCCACATCTAC |
|  | gB-R | GTCCGTGAAGCGGTTCGTGAT |

Table S10. Primers used for RT-qPCR assay.

| Primer | primer sequence (5'to3') |
| --- | --- |
| β-actin-F | GGACTTCGAGCAGGAGATGG |
| β-actin-R | AGGAAGGAGGGCTGGAAGAG |
| IFN-α-F | CCCCTGTGCCTGGGAGAT |
| IFN-α-R | AGGTTTCTGGAGGAAGAGAAGGA |
| IFN-β-F | CCAGCAGATCTTCGGCATTC |
| IFN-β-R | CAGGTCATCCATCTGCCCATC |
| ISG15-F | CGTGCAAGCTGACCAGTTCTG |
| ISG15-R | TACACGGTGCACATAGGCTTG |
| OAS1-F | AAGTTGTGAAGGGTGGCTCC |
| OAS1-R | TCGGAGCTGAAGCTGATCCTC |
| MX1-F | CAGAGGCAGCGGAATTGTG |
| MX1-R | AATCTCGCTGTCCCGGTAAC |
| PKR-F | CAATTGGCTGCCAAACAGGC |
| PKR-R | GCTTTGGATGTCACTGCTCG |
